# Compositional Tuning of Mixed Chromium Monopotassium Phosphates by Ni^2+^/Co^2+^ Substitution for Energy Storage Applications

**DOI:** 10.1021/acs.inorgchem.5c05545

**Published:** 2026-05-28

**Authors:** Zaineb Mighri, Roxana Elena Patru, Ameen Uddin Ammar, Lucia Nicoleta Leonat, Habib Nasri, Aurelian Catalin Galca, Arpad Mihai Rostas

**Affiliations:** † Laboratory of Complex Heterostructures and Multifunctional Materials (HeCoMat), 27266 National Institute of Materials Physics, Atomistilor 405A, Magurele 077125, Romania; ‡ Laboratory of Physical Chemistry of Materials, Faculty of Sciences of Monastir, 199824 University of Monastir, Avenue de l’Environnement, Monastir 5019, Tunisia; § 61787 National Institute for Research and Development of Isotopic and Molecular Technologies, Donat 67-103, Cluj-Napoca 400293, Romania; ∥ International Centre for Advanced Training and Research in Physics, Atomistilor 409, Magurele, Ilfov 077125, Romania

## Abstract

This study synthesized a solid-solution series of KCo_1–*x*_Ni_*x*_Cr_2_(PO_4_)_3_ phosphates with the α-CrPO_4_-type three-dimensional framework, in which edge-sharing CrO_6_ octahedra and PO_4_ tetrahedra create tunnels hosting K^+^ cations. Systematic Ni^2+^/Co^2+^ substitution was used to tune dielectric and charge-transport properties. All compounds showed stable paraelectric behavior; increasing Co content enhanced polarizability and dielectric permittivity while maintaining low dielectric loss. When used as electrode materials in graphite-based supercapacitors, higher Co content led to improved electrochemical performance, with the Co1Ni0 composition delivering a specific capacitance of 447 F/g, an energy density of 48.18 Wh/kg, and a power density of 1752 W/kg. The results demonstrate that Ni^2+^/Co^2+^ substitution is an effective strategy for designing advanced supercapacitor electrodes that combine high charge-storage capacity (due to increased permittivity) with improved power capability (due to higher conductivity).

## Graphical abstract

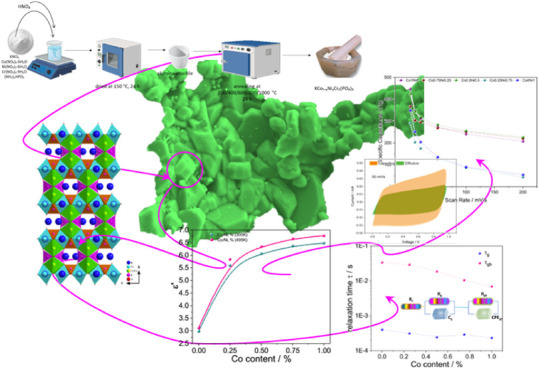

## Introduction

Recent structural and electrochemical studies on α-CrPO_4_ type phosphates underscore how their unique three-dimensional (3D) framework, comprising edge-shared CrO_6_ octahedra and PO_4_ tetrahedra in the *Imma* orthorhombic space group, forms a rigid skeleton with tunnel-like channels capable of accommodating guest cations.^1^ A prime example is NaCoCr_2_(PO_4_)_3_, synthesized via a solid-state reaction. Rietveld refinement reveals that Cr atoms totally occupy M(1) sites, whereas M(2) sites are occupied by Cr and Co atoms. Binuclear octahedra with shared edges arranged in 2D layers are then formed, interconnected with chains of CrO_6_–PO_4_ units to build a 3D network hosting Na^+^ in intersecting tunnels.^2^ This architectural motif demonstrates how substituting Cr^3+^ ions with Co^3+^ ions (balanced by Na^+^) preserves mechanical stability while structurally integrating redox-active and conductive centers.

Parallel investigations of α-Fe_1–*x*_Cr_*x*_PO_4_ (e.g., α-Fe_0.75_Cr_0.25_PO_4_) demonstrate that Cr incorporation into the α-CrPO_4_ structure yields a porous, oriented morphology (“rugby-ball”) and supports reversible Li^+^ intercalation with high discharge capacity (88 mAh/g) and outstanding cycling stability (98.5% retention over 250 cycles)^3^ affirming the structural integrity and electrochemical utility of Cr-stabilized α frameworks.

In the realm of Co–Ni phosphate nanostructures, ultrathin (Ni,Co)_3_(PO_4_)_2_.8H_2_O nanoslices realize exceptional pseudocapacitive performance, delivering specific capacitance up to 1128 F/g and energy density 35.3 Wh/kg attributable to facile redox reactions at the Ni/Co sites and minimal diffusion barriers in the ultralow-thickness architecture.^4^ Moreover, the redox flexibility of cobalt confers considerable catalytic relevance to Co sites embedded in open-framework structures.^5^ Furthermore, this d-block element exhibits optically accessible d-d transitions. The magnetic properties of cobalt and its sensitivity to its coordination environment have stimulated research on this topic. Nickel is particularly interesting because it occurs in different valence states, with coordination varying with the glass matrix. Doped sodium phosphate glasses exhibit optical absorption characteristics that are predominantly attributable to Ni^2+^ ions in octahedral coordination. Notably, UV bands in the 205–275 nm range are associated with charge-transfer processes involving residual impurities, such as iron contaminants. Within the visible spectrum, absorption peaks at around 420 and 780 nm correspond to intra-d-shell electronic transitions, confirming the presence of Ni^2+^ ions and their octahedral coordination environment in the glass matrix. These spectral attributes elucidate the valence state, local symmetry, and structural integration of nickel ions within the host network. These are mainly due to the octahedral coordination of the Ni^2+^ ions.^6,7^ A dielectric studies of KM^II^Cr­(PO_4_)_2_ compounds (M = Ni, Co) reveal a moderate Maxwell–Wagner response. KNiCr­(PO_4_)_2_ exhibits stable permittivity, while KCoCr­(PO_4_)_2_ shows slightly higher values. Relaxation behavior varies with the transition metal: nickel-based phosphates display slower dielectric relaxation, whereas cobalt-based analogs support faster polarization and depolarization dynamics. These differences highlight the influence of cation substitution on dielectric performance.^8^

Furthermore, the integration of potassium ions K^+^ in contrast to Na^+^ or Li^+^ offers compelling benefits: potassium’s abundance ensures low cost. At the same time, its electrochemical potential (−2.93 V vs SHE) is close to lithium’s (−3.04 V), enabling similarly high cell voltages and energy density.^9,10^ Despite its larger ionic size, K^+^ exhibits the smallest Stokes radius (3.6 Å) in common electrolytes, translating to lower desolvation energy, faster diffusion, and superior ionic conductivity compared to Li^+^ and Na^+^.^11,12^ Additionally, K^+^ does not alloy with aluminum, enabling the use of lightweight Al current collectors, thereby providing additional cost and manufacturing advantages.^13,14^

Crucially, potassium’s role in such frameworks is gaining attention. In KCo_*x*_Ni_1–*x*_PO_4_.H_2_O microplates, K^+^ lodged in the phosphate matrix enhances specific capacitance (227 F/g at 1.5 A/g), rate performance, and retention (82% after 5000 cycles) compared to (NH_4_)^+^ analogues, benefits likely attributed to the stabilizing presence of K^+^ in structural tunnels, which supports both ion conduction and mechanical resilience.^15^

Moreover, in carbonized hexagonal KCoPO_4_, the fluffy morphology coupled with K^+^ in the tunnel-like structure enabled a high pseudocapacitive response (725 F/g) and exceptional asymmetric supercapacitor energy density (121 Wh/kg with a high power density of 6945 W/kg in the potential window of 1.5 V in 2 M KOH electrolyte, emphasizing the synergistic charge-storage from Co^2+^/Co^3+^ redox and K-mediated structural dynamics.^16,17^ On the other hand, Ni^2+^ ions, when present in a material, enhance electronic conductivity by facilitating orbital overlap and electron delocalization, enabling rapid charge transfer. This synergy manifests spectacularly in NiCo_2_O_4_ based systems-spinel electrodes that display the highest specific capacitance of 1254 F/g at a current density of 2 A/g.^18^

Altogether, these findings reveal how Cr–Ni/Co interconnections in the α-CrPO_4_-type lattice, via shared octahedral edges and bridging phosphate groups, enhance electronic coupling and redox accessibility, while tunnel-embedded K^+^ acts both as a structural stabilizer and a charge-balancing conductor. In summary, this structural-electrochemical synergy yields materials that deliver high specific capacitance, elevated energy density, and robust cyclic stability, positioning them as powerful platforms for designing next-generation supercapacitor electrodes and potassium-ion battery materials.

For all these reasons, and because among many existing phosphates, those containing chromium are just a few, special attention is given to the synthesis and characterization of new mixed Ni/Co substituted α-CrPO_4_ -type phosphates with KCo_1–*x*_Ni_*x*_Cr_2_(PO_4_)_3_ are conducted. In this study, the influence of substituting Co^2+^ ions with smaller Ni^2+^ ions on the electrical properties of the phosphate materials is evidenced. Following a thorough morpho-structural analysis, the prepared materials were used as electrodes in asymmetric supercapacitors (SCs), demonstrating promising performance for energy storage applications.

## Materials and Methods

### Synthesis Procedure

Crystalline powders belonging to the solid solution KCo_1–*x*_Ni_*x*_Cr_2_(PO_4_)_3_ (with x = 0.00; 0.25; 0.50; 0.75; 1.00), abbreviated Co1Ni0, Co0.75Ni0.25, Co0.50Ni0.50, Co0.25Ni0.75 and Co0Ni1, were prepared by a conventional solid-state reaction, starting from a stoichiometric mixtures of nitrate, and phosphate reactants of analytical grade KNO_3_ (Fluka, 99%), Co­(NO_3_)_2_.6H_2_O (Fluka, 99%), Ni­(NO_3_)_2_.6H_2_O (Fluka, 99%), Cr­(NO_3_)_3_.9H_2_O (Fluka, 99%), and (NH_4_)_2_HPO_4_ (Merck, 99%) as summarized in Figure 1. This mixture was dissolved by adding an aqueous nitric acid solution, stirred continuously, then air-dried for 24 h in an oven at 150 °C to remove the remaining water. The dry compounds were placed in a platinum crucible and thermally treated with successive annealing at 200, 400, 600, 800, and 1000 °C for 24 h in a muffle oven. Gaseous nitrogen dioxide and ammonia were removed during heating cycles and interspersed with several intermediate grindings in an agate mortar.

**Figure 1:**
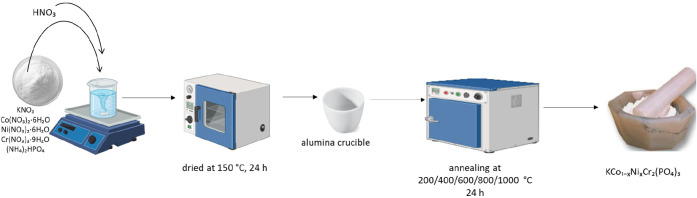
Schematic representation of the synthesis procedure used to synthesize the KCo_1–*x*_Ni*_x_* Cr_2_(PO_4_)_3_ compounds.

### Morpho-Structural Analysis

X-ray powder diffraction (**XRD)** was measured at room temperature using a Bruker D8 Advance diffractometer equipped with a dichromatic Cu anticathode (Kα_1_ = 1.5406 Å and Kα_2_ = 1.444 Å) as the X-ray source and operating in a Bragg–Brentano geometry. Measurements were made in the 2θ angular range from 12° ≤ 2θ ≤ 85°, with an angular step size of 0.02°, and an integration time of 3 s per measurement point.

The chemical composition was analyzed using an energy-dispersive X-ray (**EDX**) detector at an accelerating voltage of 15 kV by field-emission scanning electron microscopy (**SEM**) on a Zeiss Gemini 500 instrument. The samples were prepared by depositing a small uniform amount of powder on slabs using adhesive carbon disks.

Fourier transform infrared (**FTIR**) spectroscopy was employed in the attenuated-total-reflectance (ATR) mode, a well-established technique for analyzing and identifying materials’ molecular arrangement and chemical structure. A PerkinElmer Spectrum BX II spectrometer equipped with a Pike-MIRacle ATR head with a ZnSe-diamond crystal plate was used. The IR absorption spectra were recorded in the 50–4000 cm^–1^ range.

Raman spectroscopy was performed at room temperature in the 100–1400 cm^–1^ range with a backscatter configuration using a Horiba Jobin-Yvon LabRAM HR Evolution spectrometer equipped with a confocal microscope and a He–Cd laser operating at 325 nm, using a 40× Olympus objective, with adjusted intensity to avoid laser-induced heating on the surface of the samples.

Electron paramagnetic resonance (EPR) spectroscopy measurements were performed at room temperature with a continuous-wave X-band Bruker E500 EPR spectrometer fitted with a Bruker X-SHQ 4119HS-W1 X-band resonator operating at microwave frequencies of 9.881 GHz.

### Electric Measurements

The sintered ceramic discs were polished and coated on both faces with silver paste, forming parallel-plate electrodes for electrical characterization. Dielectric measurements were performed in a vacuum using a computer-controlled HIOKI IM3536 precision LCR meter. A sinusoidal excitation of 1 V was applied while sweeping the frequency from 10^2^ to 10^6^ Hz. The thermal protocol consisted of programmed cooling from 495 to 50 at 1 K/min, with thermal stability maintained during acquisition within a ± 0.03 K error range.

### Supercapacitor Applications

The electrochemical performance of the Ni/Co phosphate-based sample was tested by preparing a mixture of 2.3 mg of the synthesized sample and 3 mg of graphite as the electrode material in a symmetric supercapacitor cell. The supercapacitor device has circular stainless-steel bolts on both sides that serve as current collectors. The areal mass loading of the electrodes on this device is approximately 4.7 mg/cm^2^ over an area of 1.13 cm^2^. 100 μL of 1 M KOH as electrolyte was used, and a glass fiber separator was placed between the electrodes. Graphite and the synthesized sample were mixed and then deposited onto the current collector surface without a binder to evaluate the electrochemical contribution of the active electrode material exclusively. A schematic illustration of the supercapacitor cell is provided in Scheme S1 for better visualization. The electrochemical performance of the materials was tested with a BioLogic VMP 300 electrochemical workstation. Galvanostatic cycling with potential limitation (GCPL), potentiostatic electrochemical impedance spectroscopy (PEIS), and cyclic voltammetry (CV) were used to evaluate the performance of the supercapacitor devices. GCPL tests were performed at a current density of 0.8 A/g. The amplitude of the AC signal (10 mV) in the frequency range of 10 mHz to 1 MHz was the parameter for the PEIS measurements. CV measurements were performed over a voltage window of 0–1 V at scan rates of 2, 10, 20, 50, 100, and 200 mV/s.

## Results and Discussion

### Morpho-Structural Properties Determination

SEM images of the KCo_1–*x*_Ni_*x*_Cr_2_(PO_4_)_3_ compounds (Figure 2a-e) reveal the formation of large, angular agglomerates composed of smaller, rounded primary particles. This tendency toward agglomeration becomes more pronounced with increasing nickel content, suggesting a correlation between particle morphology and the degree of substitution.

**Figure 2:**
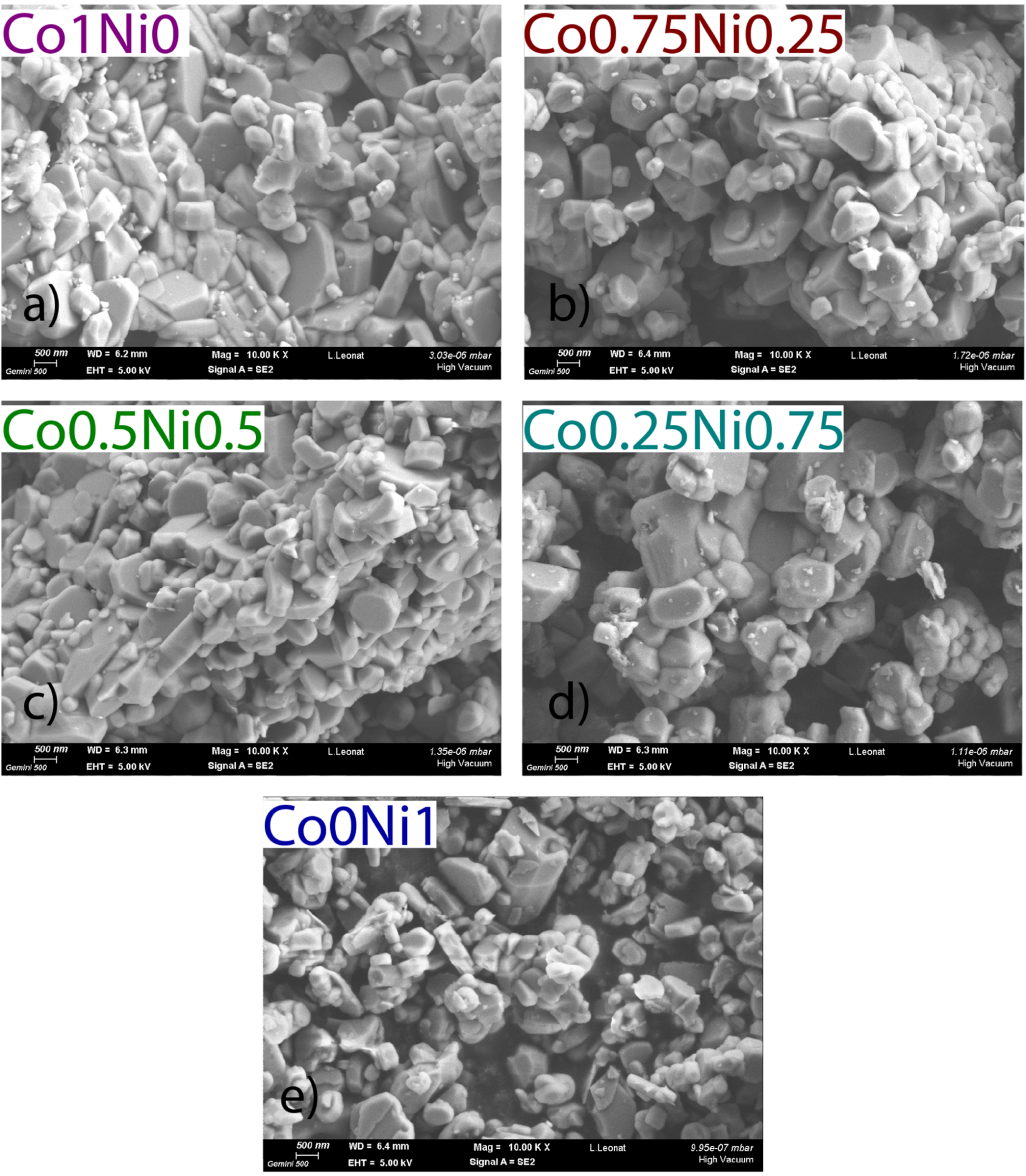
SEM micrographs of the Co1Ni0 (a), Co0.75Ni0.25 (b), Co0.5Ni0.5 (c), Co0.25Ni0.75 (d), and Co0Ni1 e).

This behavior may be attributed, on one hand, to the intrinsic magnetic character of chromium, which is present in excess relative to the divalent metal cations M^2+^, with a fixed stoichiometric ratio of M^2+^:Cr^3+^ = 1:2 across all samples. On the other hand, the weak van der Waals forces acting between the fine particles may also contribute to their aggregation into larger structures.

EDX results presented in Figure S1 confirm the presence of all expected elements in the analyzed samples and indicate good compositional homogeneity (Table 1). Moreover, the experimentally determined molar ratios closely match the theoretical values, demonstrating reliable control over the material synthesis process.

**Table 1:** Experimental Molar Ratios of Each Element in the KCo_1–*x*_Ni*_x_* Cr_2_(PO_4_)_3_ Compounds (with 0 ≤ *X* ≤ 1)

Atom %	Co1Ni0	Co0.75Ni0.25	Co0.5Ni0.5	Co0.25Ni0.75	Co0Ni1
K	5.26	5.29	5.27	5.27	5.01
Cr	10.44	11.91	11.30	9.88	10.89
Co	5.06	4.03	5.51	0.98	-
Ni	-	1.42	5.53	3.57	5.25
P	15.35	14.43	14.98	13.81	14.54
O	54.28	55.11	60.15	60.95	57.85

Crystallochemical measurements (Figure 3a) carried out on the A_3_PO_4_-M_3/2_^II^(PO_4_)_2_-M^III^PO^4^ system where A, M^II^ and M^III^ are respectively an alkali, a divalent element and a transition metal, revealed two limiting compositions with KM^II^Cr_2_(PO_4_)_3_ (where M^II^ = Co, Ni) as well as a series of solid solution compounds with KCo_1–*x*_Ni_*x*_Cr_2_(PO_4_)_3_ (where x = 0.25; 0.50; 0.75). To investigate the structural impact of substituting cobalt with nickel within this solid solution, a preliminary examination of the diffractograms, based mainly on data from the High-Score Plus database,^19^ an automatic search of the PDF-ICDD database, followed by structural Rietveld refinement using the TOPAS program^20^ (see Figures 3b, 3c, and S2), confirmed that the synthesized powder materials crystallize in an orthorhombic system with space group *Imma* (No. 74). The diffraction patterns indicate a single-phase nature and an isotypic relationship with the structural type α-CrPO_4_. The atomic coordinates are similar to the previously reported NaV_3_P_3_O_12_ compound.^21^ The crystallographic data and the goodness of refinement from X-ray data of the solid solution of our prepared materials are gathered in Table S1.

**Figure 3:**
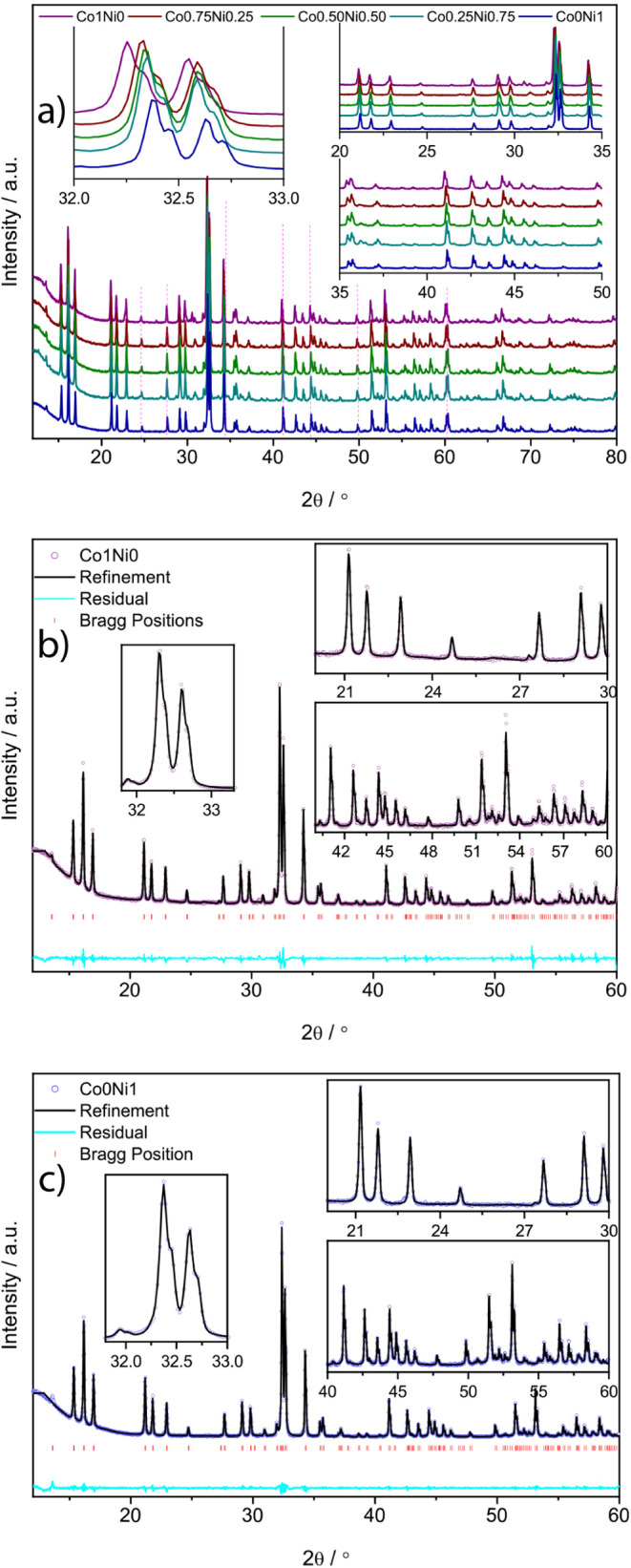
XRD comparison (a) of the KCo_1–*x*_Ni*_x_* Cr_2_(PO_4_)_3_ compounds, and Rietveld analysis results of Co1Ni0 (b), and Co0Ni1 (c).

To gain a deeper insight into the structural arrangement of the chains within the framework, we will take the crystal structure of the two limiting compositions Co1Ni0 (Figure 3b) and Co0Ni1 (Figure 3c) as a representative example of all the samples mentioned above. Their asymmetric unit comprises nine distinct atomic positions: one occupied by potassium K^+^ ions named site X(1) located at the 4*e* positions in the Wyckoff notation, one totally occupied by chromium Cr^3+^ ions named M(1) site, located at the 4*a* positions in the Wyckoff notation, forming an octahedral environment with the O atoms. One dimeric M(2) site shared between Cr^3+^ and M^2+^ divalent metal ions (where M^2+^ = Co, Ni), located at the 8*g* positions in the Wyckoff notation, two for phosphorus P(1) and P(2) with, respectively, 4*e* and 8*g* positions in the Wyckoff notation, and four for oxygen O. However, in terms of solid solution compounds, it should be noted that the M(1) site is exclusively occupied by chromium Cr^3+^ ions. Whereas, the site M(2) is shared between chromium Cr^3+^ and divalent metal ions M^2+^ in all the samples mentioned above. The atomic coordinates, site occupancies, and isotropic thermal displacement parameters of the synthesized phosphates are summarized in Table S2.

The spatial distribution of Cr^3+^ and M^2+^ ions at the M(1) and M(2) sites is identical in all the structures. Similarly, the K^+^ ions exhibit the same distribution, occupying exclusively a single type of alkali site located within intersecting structural channels. All atomic sites lie on special crystallographic positions along 2-fold axes or inversion centers except for O(21) and O(22), which occupy general positions within the *Imma* space group.

The three-dimensional anionic framework is constructed from two different types of octahedra M(1)­O_6_ and M(2)­O_6_ and two different types of tetrahedra P(1)­O_4_ and P(2)­O_4_. The M(1)­O_6_ octahedra are spatially isolated, while the M(2)­O_6_ units are edge-sharing, forming the M(2)_2_O_10_ a pair of edge-sharing octahedra. The spatial arrangement and connectivity of these polyhedral units are depicted in Figure 4a-c. Each M(1)­O_6_ octahedron (Table 2) shares all six of its vertices with surrounding phosphate tetrahedra, four of the P(1)­O_4_ type and two of the P(2)­O_4_ type. Notably, four vertices are connected with four adjacent M(2)_2_O_10_ a pair of edge-sharing octahedra.

**Figure 4:**
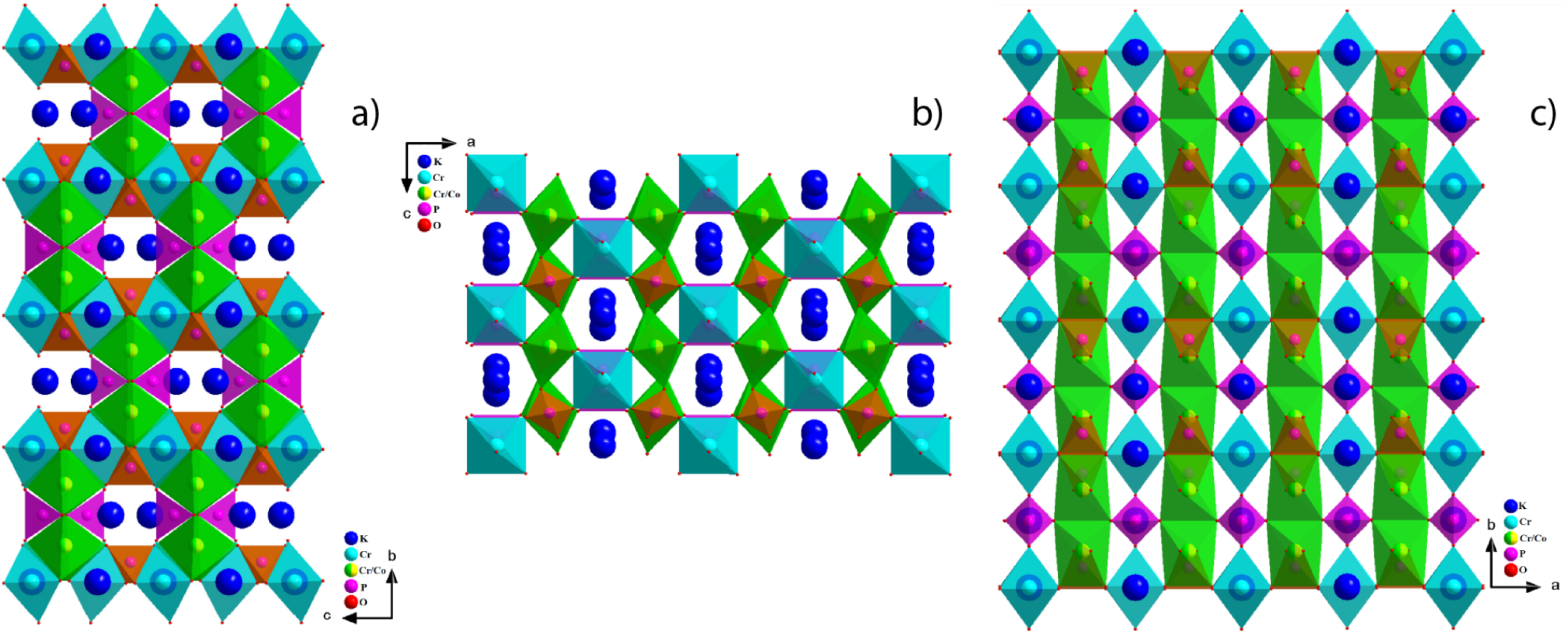
Polyhedral units’ spatial arrangement and connectivity in the *bc* (a), *ac* (b), and *ab* (c) planes for Co1Ni0 sample.

**Table 2:**
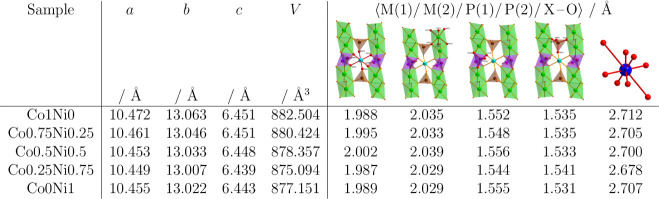
Lattice Parameters, Volume, and Main Interatomic Distance of the Co_1–*x*_Ni*_x_* (X= 0, 0.25, 0.5, 0.75, and 1) Compounds

Each M(2)­O_6_ octahedron (Table 2) shares two vertices with two P(1)­O_4_ tetrahedra and two opposite edges, one with a P(1)­O_4_ tetrahedron and the other with the adjoining M(2)­O_6_ octahedron to form the dimer M(2)_2_O_10_. The oxygen atoms constituting this shared edge are further linked to the P(2)­O_4_ tetrahedra, contributing to the overall polyhedral connectivity.

This 3-D network gives rise to two distinct types of tunnels: one with a rectangular cross section extending along the [100] direction (Figure 4a), and another with a hexagonal cross section aligned along the [010] direction (Figure 4b). The K^+^ ions are located at the intersection points of these two types of channels, occupying strategic positions within the framework.

The principal interatomic distances for the two limiting compounds and the solid solution compounds are summarized in Table S3. The six oxygen atoms coordinating the M(1) site form an M(1)­O_6_ octahedron, composed of two distinct sets of bonds. Four oxygen atoms lie in a square planar arrangement around the metal center, with M(1)-O bond lengths of 1.988(3) Å for Co1Ni0 and 1.984(2) Å for Co0Ni1. The remaining two oxygen atoms occupy axial positions above and below this square plane, at distances of 1.993(3) Å and 2.000(1) Å, for Co1Ni0 and Co0Ni1, respectively. The near-equal bond lengths in all six positions reflect the high symmetry and minimal distortion of the M(1)­O_6_ octahedron, as confirmed by the low variation among the M(1)-O distances.

The M(2)­O_6_ octahedra are slightly more distorted, because of the mixture from Cr^3+^ and M^2+^ ions, exhibiting three distinct M(2)-O bond pairs: 2.005(3) Å, 2.041(2) Å, and 2.058(3) Å for Co1Ni0, and 2.011(2) Å, 2.035(2) Å, and 2.042(3) Å for Co0Ni1.

The average M-O distances presented in Table 2, further reflect this distinction: the M(1)-O bond lengths average 1.988(3) Å for Co1Ni0 and 1.989(2) Å for Co0Ni1, while the M(2)-O distances average 2.035(4) Å and 2.029(4) Å, for Co1Ni0 and Co0Ni1 respectively, consistent with previously reported M-O bond lengths for Cr^3+^ in NaCoCr_2_(PO_4_)_3_, and for six-coordinate Ni^2+^ in NaNiCr_2_(PO_4_)_3_.^2^

The phosphate units in both compounds exhibit two types of P–O bonds, associated with the P(1)­O_4_ and P(2)­O_4_ tetrahedra. The main interatomic distances are presented in Table S3. For the P(1)­O_4_ group, bond lengths range between 1.541(2) and 1.563(2) Å in Co1Ni0, and between 1.547(2) and 1.562(2) Å in Co0Ni1. The P(2)-O bond lengths span 1.507(2)–1.562(4) Å in Co1Ni0, and 1.484(2)–1.578(4) Å in Co0Ni1. These values are in good agreement with the typical P–O bond length of 1.537 Å reported by^22^ for anhydrous monophosphates, confirming the structural integrity and absence of hydrogen bonding in these frameworks.

The K^+^ cations occupy a single-type site at the intersection of the crossing tunnels. Its environment has been determined by considering all the cation-oxygen distances lower than the *L*_max_ = 3.33 Å limits suggested by^23^ with eight coordination and ranges from 2.642(2) to 2.763(3) Å for Co1Ni0 and between 2.635(4) and 2.778(2) Å for Co0Ni1.

The valence states (BVS) of all cationic sites were determined using the empirical bond valence method developed by Brown and Altermatt^24^ $V_{j}=\underset{i}{\sum }\exp\left(\frac{r_{0}-r_{ij}}{B}\right)$ , where *Vj* is the oxidation state of the cation *j*, *r*_0_ describes the cation–anion distance (2.132 Å for K^+^, 1.692 Å for Co^2+^, 1.654 Å for Ni^2+^, 1.724 Å for Cr^3+^, and 1.617 Å for P^5+^), *r*_*ij*_ is the distance between the *i* and *j* atoms and *B* = 0.37. The summation of individual bond valences around each cationic center yields its oxidation state. The calculated valences (see Table S3) are consistent with the expected formal oxidation states of each cation M^2+^, Cr^3+^, and P^5+^, thereby validating the structural model. The only exception was the calculated bond valence for K^+^ with 1.68 in Co1Ni0 and 1.72 in Co0Ni1. These values are notably higher than the expected value of 1 for K^+^. This discrepancy may be attributed to the relatively large ionic radius of K^+^ relative to the tunnel dimensions, resulting in unusually short K–O bond distances.

The evolution of the α-CrPO_4_ unit cell parameters (*a*, *b*, and *c*) and the corresponding unit cell volume V as a function of the substitution rate are illustrated in Table 2 and Figure 5. *a* and *b* decrease in all the prepared phosphates, a trend that correlates with the ionic radius of each divalent cation (r­(Co^2+^) = 0.745 Å), (r­(Ni^2+^) = 0.69 Å) located in an octahedral site.^25^ The same is observed for the *c* parameter, which shows a slight decrease with substituting cations of lower ionic radius, relating to the size of the monovalent A^+^ cations (A = Na, K, Rb) located in the tunnels formed in the structure and which are parallel to the *c*-axis. This can be explained by the fact that only the K^+^ cations occupy the tunnels in all the compounds.

**Figure 5:**
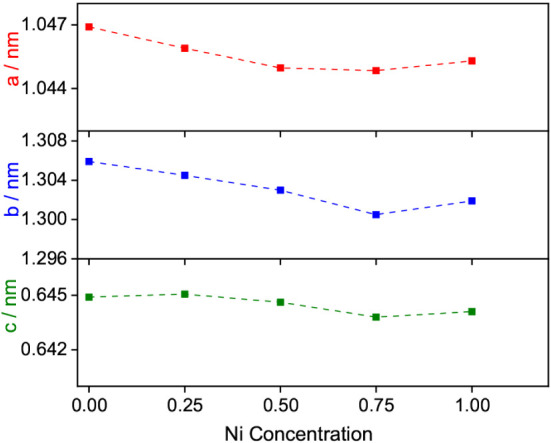
Lattice parameters (*a*, *b*, and *c*) variation with Ni/Co substitution.

The unit cell volume decreases nearly linearly from 882.504 Å^3^ for Co1Ni0 to 875.094 Å^3^ for Co0.25Ni0.75 consistent with the substitution of larger Co^2+^ (r­(Co^2+^) = 0.745 Å) by smaller Ni^2+^ (r­(Ni^2+^) = 0.69 Å) ions occupying equivalent coordination environments. However, the Co0Ni1 end-member exhibits a slight positive deviation from this trend, likely due to subtle framework relaxation and reorganization of the MO_6_–PO_4_ 3D tunnel network upon complete Ni substitution.

The crystallites size was calculated using Scherrer’s empirical method: *D* = (*Kλ*)/(βcosθ), where *D* is the size of the crystallites, *K* is Scherrer’s constant (*K* = 0.9), is the full width at half-maximum (fwhm, in radians), and λ is the wavelength of the X-rays used λ = 0.15406 nm), and θ is the peak position. As is known, XRD peak broadening primarily arises from crystallite size and microstrain. The Scherrer method estimates crystallite size using the fwhm, which reflects only size-related broadening. In contrast, the Williamson-Hall method uses the peak integral breadth, enabling distinction between size and strain effects by analyzing peak area and height. As a result, the microstrain calculated using the Williamson-Hall model using the corresponding formula: ϵ = (β/(4tanθ), where ϵ is microstrain, θ is the peak position, and β is the full width at half-maximum.^26^ The obtained crystallite size and microstrain values are regrouped in Table S4. These values, ranging from 177.8 to 202 nm, indicate the formation of submicron nanocrystalline domains.

### FT-IR and Raman Spectroscopy Characterization

Comparing the IR absorption bands (Figure 6a) associated with PO_4_ groups, depending on the nature of the divalent element, reveals that this difference could cause the polarizing power of the divalent cation M^2+^. In infrared spectroscopy, a decrease in the frequency of a valence vibration band indicates a reduction in the bond strength between the two atoms involved. As the polarizing power increases (i.e., as the size decreases and the charge increases), the covalence of the M-O bond increases and the ionic character of the antagonistic P–O bond is reinforced. This implies a shift of the IR absorption bands toward lower wave numbers. In the series of compounds studied, the charge of the divalent ion remains constant, meaning that the evolution of the IR spectra depends solely on its size. Indeed, a slight shift of the IR absorption bands of the PO_4_ groups toward lower wave numbers is observed when moving from Co (r­(Co^2+^) = 0.745 Å) to Ni (r­(Co^2+^) = 0.69 Å). Furthermore, the assignment of IR absorption bands to the vibration modes of PO_4_ tetrahedra in all cases is consistent with the presence of two crystallographically independent phosphate groups, confirming the structural determination.

**Figure 6:**
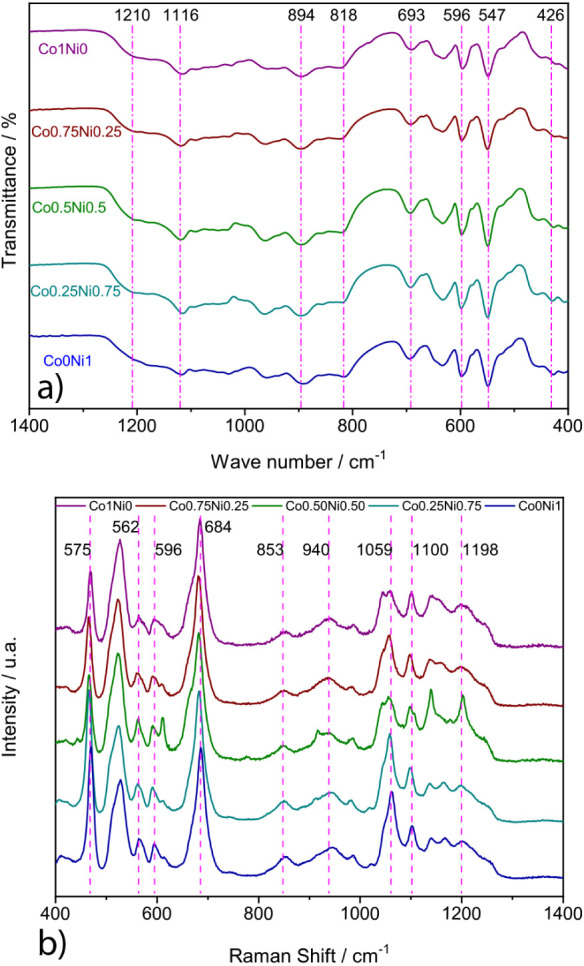
FT-IR (a) and Raman (b) spectra of the KCo_1–*x*_Ni*_x_* Cr_2_(PO_4_)_3_ compounds.

The bands observed at 1210–1116 cm^–1^ and 894–818 cm^–1^ are attributed to asymmetric (ν_3_) and symmetric (ν_1_) P–O stretching, respectively. This finding is consistent with previous studies of.^27^ The bands observed between 693 and 426 cm^–1^ can be attributed to the asymmetric (ν_4_) and symmetric (ν_2_) O–P–O angular deformation vibrations, as well as the vibrations of the MO_6_ octahedra.^28^

To further consolidate our structural analysis, we also performed Raman spectroscopy on the various compounds studied by IR at room temperature in the 100–1400 cm^–1^ range. Figure 6b shows the Raman spectra of the KCo_1–*x*_Ni_*x*_Cr_2_(PO_4_)_3_ (with 0 ≤ *x* ≤ 1) compounds. The bands observed between 1198 and 1059 cm^–1^ correspond to the asymmetric elongation of the P–O bonds.^29,30^ Those appearing between 940 and 853 cm^–1^ are assignable to the symmetric stretching of the same type of bond. The bands between 684 and 575 cm^–1^ may be related to the asymmetric (ν_4_) and symmetric (ν_2_) O–P–O angular deformation vibrations.^29,31^

### Electron Paramagnetic Resonance Spectroscopy Characterization

Temperature-dependent EPR measurements were performed between 150 and 360 K for the two limiting compositions, Co1Ni0 and Co0Ni1, revealing distinct spectral features. For Co1Ni0 (Figure 7a), the spectrum exhibits a single, intense, and narrow signal, characteristic of paramagnetic ions in an octahedral coordination environment, with an effective g-factor (*g*_eff_) of approximately 1.975. In contrast, the Co0Ni1 sample (Figure 7b) displays a broader and less intense signal, with a slightly lower (*g*_eff_) of 1.96.

**Figure 7:**
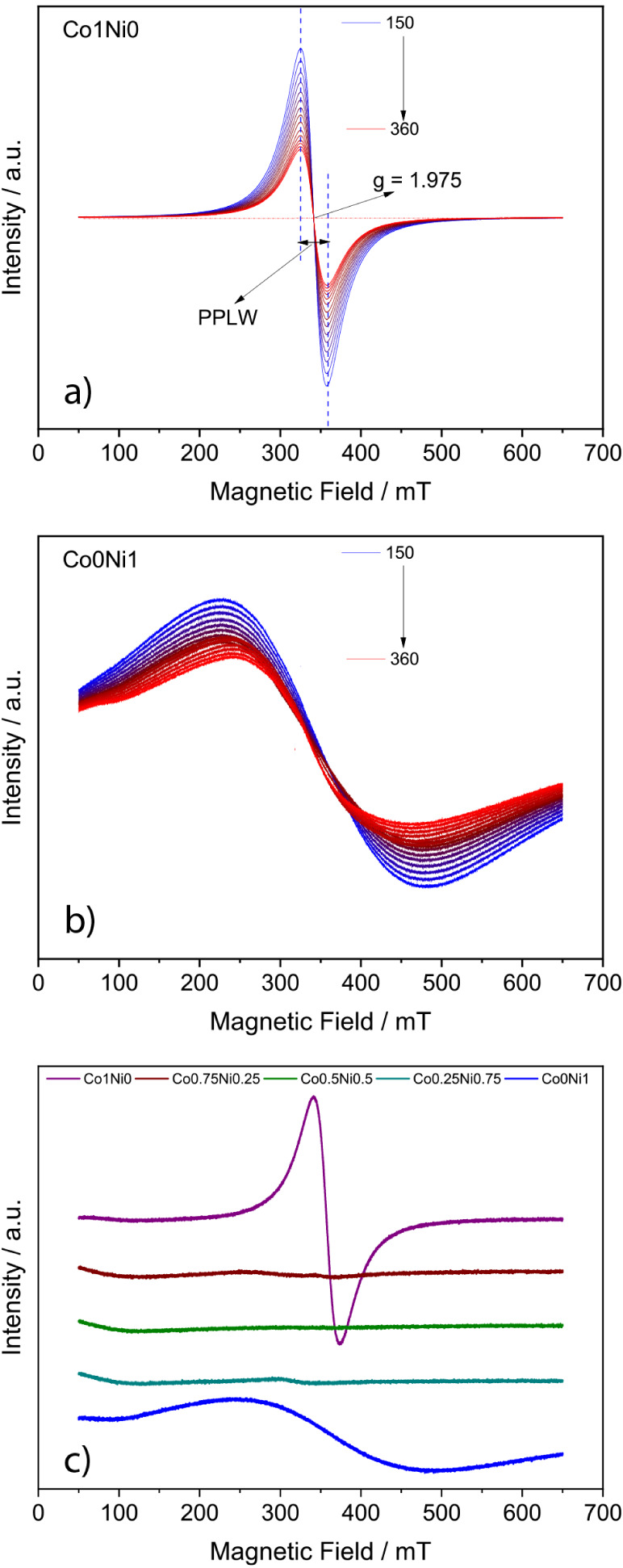
EPR spectra of the Co_1_Ni_0_ (a) and Co_0_Ni_1_ (b) samples with temperature dependency, respectively, and the mixed Ni/Co compounds measured at room temperature (c).

These differences in spectral characteristics may arise from local magnetic interactions, either through the formation of Cr^3+^ - M^2+^ (where M^2+^ = Co^2+^, Ni^2+^) ion pairs or through the presence of paramagnetic clusters in which spins are antiferromagnetically coupled. In the first case, magnetic coupling between neighboring Cr^3+^ and M^2+^ ions can alter the local magnetic environment, influencing the EPR signal’s shape and intensity. In the second case, antiferromagnetic interactions between adjacent paramagnetic ions may lead to partial spin compensation, thereby reducing the net magnetic moment and altering the EPR response.

Alternatively, in the Co1Ni0 composition, the observed signal may result specifically from Cr^3+^ - Co^2+^ pairs, which appear to enhance magnetic interactions.^32^ In contrast, substituting Ni^2+^ for Co in Co0Ni1 appears to reduce these interactions, leading to weaker coupling between paramagnetic centers and, consequently, a broader, less intense signal. Notably, no EPR signal attributable to isolated Cr^3+^ ions was detected, even though Cr^3+^ belongs to the d^3^ configuration, which has three unpaired electrons (S = 3/2) and typically gives rise to a highly anisotropic spectrum characterized by *g*_∥_ ≈ 4.5 and *g*_⊥_ ≈ 1.9.

At room temperature, both the intensity and peak-to-peak line width (*H*_*PP*_) of the EPR signals for the mixed phosphate compositions Co0.75Ni0.25, Co0.5Ni0.5, and Co0.25Ni0.75 decrease progressively with increasing nickel content (Figure 7c). This trend suggests a direct correlation between Ni^2+^ substitution and the weakening of the EPR response. To sum up, in the KCo_1–*x*_Ni_*x*_Cr_2_(PO_4_)_3_ (with x = 0.25; 0.5; 0.75), enhanced magnetic interactions between the spins of Cr^3+^ and Co^2+^ ions can occur. Incorporating Ni^2+^ ions perturbs this magnetic coupling, effectively diluting or disrupting the spin network. As a result, the EPR signal intensity is significantly reduced, reflecting diminished magnetic interactions and increased spin relaxation due to the presence of Ni^2+^.^32^

### Low-Field Dielectric Properties Determination

#### Temperature-Dependent Dielectric Properties

The low-field dielectric study of the Co_1–*x*_Ni_*x*_ mixed phosphates synthesized by a conventional solid-state route and consolidated into pellets allows tracing the evolution of the dielectric response and tracking how Ni^2+^/Co^2+^ substitution tunes the intrinsic polarizability and conduction-related losses. The temperature-dependent dielectric response was examined over a broad frequency range (100 Hz – 1 MHz) in terms of the real part of the permittivity (ϵ′) and dielectric losses (tanδ). Figure 8 compiles the dielectric response for the KCo_1–*x*_Ni_*x*_Cr_2_(PO_4_)_3_ compounds and shows the ϵ′(*T*) and tanδ­(*T*) evolution according to the Ni^2+^/Co^2+^ ratio variation.

**Figure 8:**
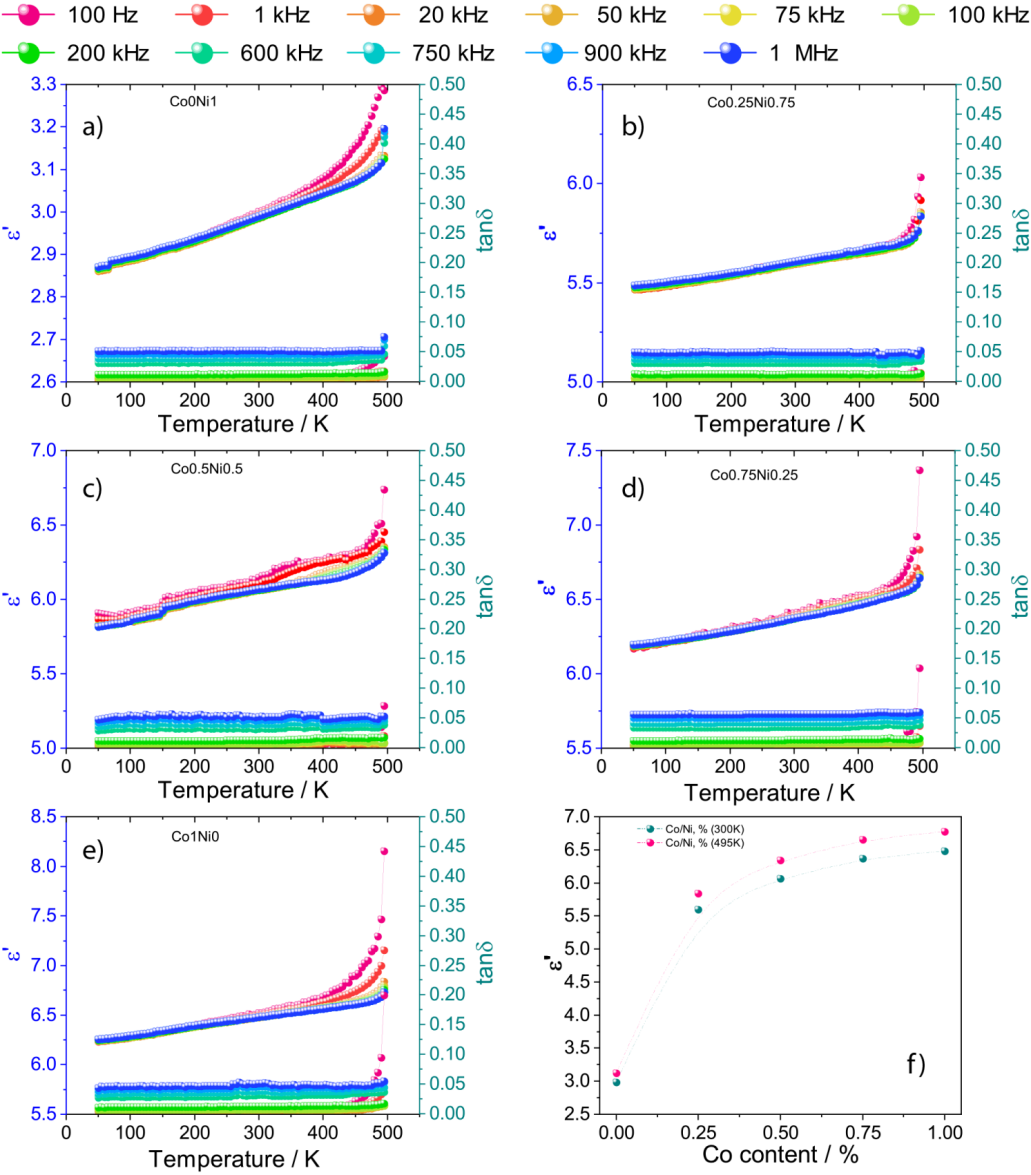
Temperature-dependent dielectric response of KCo_1–*x*_Ni*_x_* Cr_2_(PO_4_)_3_ samples (0 ≤ *x* ≤ 1): real permittivity ϵ′ (left axis) and loss tangent tanδ (right axis) vs temperature recorded at probing frequency of 102 to 106 Hz for all compositions: (a) Co0Ni1, (b) Co0.25Ni0.75, (c) Co0.5Ni0.5, (d) Co0.75Ni0.25, (e) Co1Ni0; (f) compositional dependence of ϵ′ at ambient (300 K) and at a representative high operating temperature (495 K), dashed lines are guides to the eye.

As expected for phosphate lattices, the Co–Ni compounds display a small-signal dielectric response under low field. The real part of permittivity increases smoothly, nearly linearly with the temperature for all compositions, without variation of permittivity that would indicate ferroic transitions with increasing temperature (Figure 8a-e). The Co_1–*x*_Ni_*x*_ phosphates exhibit stable, intrinsically paraelectric behavior between 100 Hz and 1 MHz, essentially dispersion-free up to 400 K across all compositions. Only a weak frequency dispersion is present below 1 kHz, in the upper end of the temperature range between 400–495 K, with ϵ′ bending upward and rising of tanδ, which is ascribed to conduction-assisted Maxwell–Wagner polarization.^33^ Dielectric losses increase slightly at low frequencies with further heating due to this polarization mechanism. At frequencies below 1 kHz, the Co0Ni1 maintains the lowest losses overall, and lowest permittivity, while with increasing Co content, a conduction-related dissipation emerges at low frequency near 400 K. For each composition, the increase of tanδ at low frequency and high temperature marks the onset of thermally activated charge transport entering the dielectric channel. The thermally activated charge transport contributes to dissipation only at high temperatures and low frequencies. At frequencies above 1 kHz, all compounds exhibit similar dielectric losses below 5% over the temperature range from 50 to 495 K, which remain stable with increasing temperature.

The intrinsic dielectric response follows the cobalt content, thus the real part of permittivity ϵ′ increases (Figure 8f). The increasing in permittivity originates from the intrinsic α-CrPO_4_ lattice effects when Ni^2+^ ions are substituted with Co^2+^ ions. Co^2+^ ionic radii are larger than Ni^2+^ ones in octahedral coordination (0.745 Å vs 0.69 Å), leading to a small expansion of the unit cell and slightly longer M(2)-O bonds with Co increasing (e.g., average M(2)-O ≈ 2.029 Å for Co0Ni1 vs 2.035 Å for Co1Ni0; unit cell volume increases from 877.67 to 882.51 Å^3^ from Co0Ni1 to Co1Ni0). Thus, the polarizing power at the M(2) site is reduced, yielding slightly longer, more distorted M-O bonds under small fields, thereby enhancing ionic polarizability and further increasing the dipolar response.

The Co-centered pairs of edge-sharing octahedra rapidly enhance the lattice polarizability of the M(2)-O_6_ connected through CrPO_4_ units and the space-charge responsiveness. The result is a steep increase in permittivity ϵ′ upon the initial small Co addition (from Co0Ni1 to Co0.25Ni0.75). Once the most effective pathways are established, further Co additions yield diminishing increments (from Co0.25Ni0.75 to Co0.75Ni0.25), approaching saturation toward Co1Ni0. A slight gap between the two selected temperatures, 300 and 495 K, is observed, reflecting the usual thermal enhancement of permittivity.

### Frequency-Dependent Dielectric Response and Dielectric Relaxation

The frequency dispersion of the complex permittivity enables separation of the intrinsic lattice contributions from thermally activated, conduction-assisted interfacial effects. Figure 9 shows the frequency dependence of the real part, ϵ′(ν), and the imaginary part, ϵ″(ν), of the permittivity for all compositions at a few selected representative temperatures of 50, 300, and 495 K. Regardless of the Co content, ϵ′(ν) is essentially frequency-invariant across the entire measured range (100 Hz – 1 MHz) (Figure 9a and 9b), and ϵ″(ν) remains below 0.4 (Figure 9d and 9e). Thus, the dominant polarization mechanisms are intrinsic to the α-CrPO_4_ structure. Such mechanisms are fast, with relaxation frequencies far above the measurement range. In this regime, charge carrier mobility and conduction-related losses are negligible.

**Figure 9:**
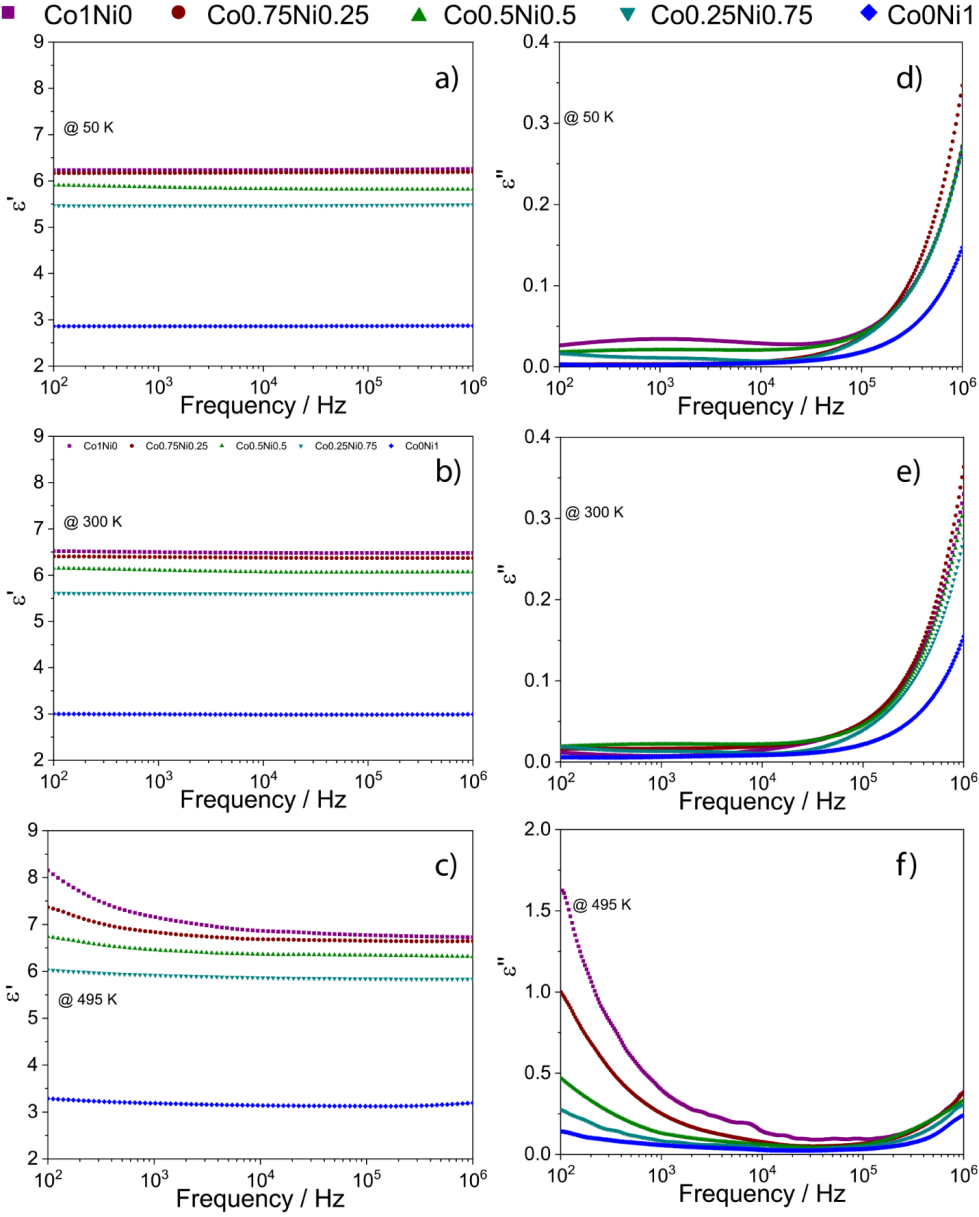
Frequency-dependent dielectric response of KCo_1–*x*_Ni*_x_* Cr_2_(PO_4_)_3_ phosphates (0 ≤ *x* ≤ 1): (a-c) real permittivity ϵ’ vs frequency, (d-f) imaginary permittivity ϵ” vs frequency at 50, 300, and 495 K for all compositions (x = 0, 0.25, 0.50, 0.75, 1).

The dielectric spectra change considerably when heated to 495 K (Figure 9c and 9f). A strong dielectric relaxation phenomenon emerges, superimposed on the conductivity effects. The real part of permittivity exhibits a pronounced dispersion, ϵ′(ν), decreasing to a constant value at high frequencies. Simultaneously, the imaginary part ϵ″(ν) sharply increases at low frequencies, following a characteristic 1/ω dependence below 1–10 kHz. This behavior is a clear signature of the contribution to dielectric loss of long-range charge transport.^34^ The stepped characteristic in ϵ′(ν) coupled with the loss peak masked by conductivity in ϵ″(ν) is typical of Maxwell–Wagner relaxation, usually associated with charge accumulation at interfaces, such as grain boundaries in polycrystalline phosphates.

The sample with higher Co amount consistently shows higher ϵ′(ν) values across the frequency range and a more prominent low-frequency dispersion at 495 K (Figure 9c and 9f). This complex behavior at high temperatures, driven by intrinsic polarizability and charge transport, requires a detailed quantitative analysis to deconvolve the underlying relaxation processes. The α-CrPO_4_-type structure of the Co_1–*x*_Ni_*x*_-based phosphates combines several polarizable units (M-O bonds connected with the pair of edge-sharing octahedra M(2)­O_6_, PO_4_ groups, K^+^, M(2) site statistically shared by Ni^2+^/Co^2+^) with different bond lengths/angles due to the Ni^2+^/Co^2+^ substitution which leads to microstructural heterogeneity at the grain boundaries. Thus, a distribution of relaxation times naturally arises, so a single-time Debye model is insufficient and cannot capture these features at the local scale. The Havriliak–Negami (*HN*) formalism that parametrizes the non-Debye type dielectric relaxation in disordered dielectrics and relaxor materials, is expressed as^35^

1 $$\epsilon \left(\omega \right)=\epsilon_{\infty }+\frac{\Delta \epsilon }{{[1+i(\omega \tau {)}^{1-\alpha_{HN}}]}^{\beta_{HN}}}$$

where ϵ­(ω) = ϵ′(ω) – *iϵ*″(ω) is the complex permittivity, Δϵ = ϵ_*S*_ – ϵ_∞_ is the relaxation strength with ϵ_*S*_ the static permittivity at zero frequency defined as the low-frequency plateau of ϵ′(ω) and ϵ_∞_ the high-frequency permittivity, while τ is the relaxation time, *i* is the imaginary unit $i=\sqrt{-1}$ , and ω is the angular frequency. The parameters α_*HN*_ (0 < α_*HN*_ < 1) and β_*HN*_ (0 < β_*HN*_ ≤ 1) account for the symmetric and asymmetric broadening of the relaxation peak.

The Havriliak–Negami model was simultaneously fitted to the full frequency dispersion of the complex permittivity with the components ϵ′(ω) and ϵ″(ω) at 495 K, and the results are presented in Figure 10.

**Figure 10:**
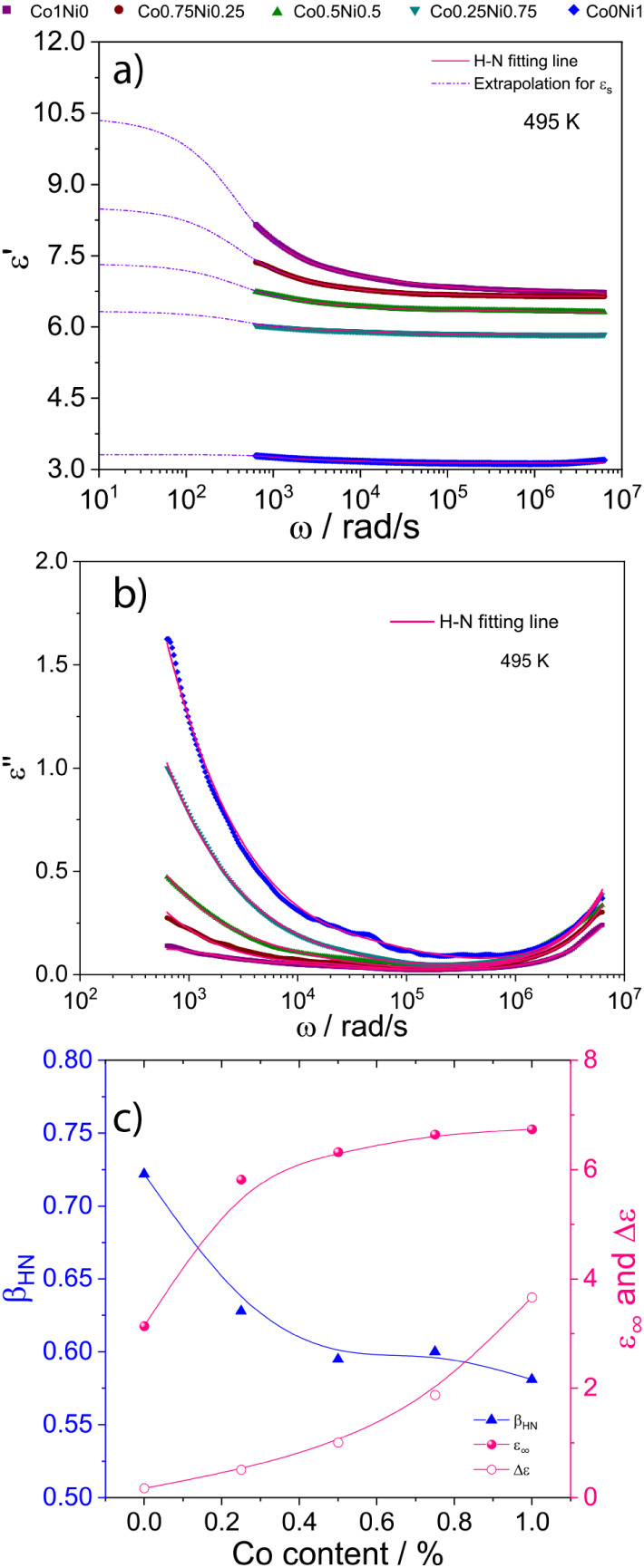
Real permittivity ϵ′(ω) (a) and imaginary permittivity ϵ″(ω) (b) with the HN fits. The asymmetric broadening factor β*_HN_* (left axis), the high-frequency permittivity ϵ_∞_, and the relaxation strength Δϵ (right axis) as a function of Co content (c).

The *HN* model accurately reproduces the ϵ′(ω) and ϵ″(ω) dispersions at the fixed high temperature of 495 K (Figure 10a and 10b) across the full frequency range between 100 Hz and 1 MHz analyzed in the angular-frequency domain (ω = 2πν). The high-frequency permittivity ϵ_∞_ was obtained directly from the *HN* model. The static permittivity at zero frequency ϵ_*s*_, which is necessary to determine the relaxation strength, was found by extrapolating the ϵ′(ω) fitted function to lim_ω→0_ ϵ′(ω) (Figure 10a). The β_*HN*_, ϵ_∞_, and ϵ_*s*_ parameters are represented as a function of the Co content (Figure 10c). For all the compounds, the 1 – *α*_*HN*_ exponent converges to unity (*α*_*HN*_ ≈ 0), thus the dielectric response at high temperatures reduces to the Cole-Davidson limit (α_*HN*_ = 0) and the asymmetric broadening of the relaxations are fully captured by the β_*HN*_parameter.^36^

In a scenario with a value of β_*HN*_ = 1, the dielectric response would approach the ideal Debye relaxation. However, subunit values obtained for the β_HN_ parameter (β_HN_ < 1) indicate a deviation from ideality, reflecting the existence of a wide distribution of time constants. As a result, in equivalent circuit modeling, using a pure capacitor is no longer appropriate. Instead, a constant phase element (CPE) connected in parallel with a resistance associated with each contribution from grains, grain boundaries, or interfaces must be employed to model the slightly nonideal behavior.^37^ The more β_HN_ deviates from 1 toward 0, the more asymmetric broadening of the relaxation at high frequencies is expected.^38^ This emerges as a fingerprint of both structural and microstructural local disorder, characterized by a distribution of fast time constants; thus, β_HN_ becomes an indicator of local disorder: the smaller β_*HN*_ is, the stronger the asymmetry. In the Co_1–*x*_Ni_*x*_ composition, β_HN_ decreases with Co^2+^ addition, showing a wider range of time constants at high frequencies, where rapid processes dominate. The progressive substitution of Ni^2+^ ions with Co^2+^ ions introduces local strain and compositional heterogeneity in the M(2) vicinity and at grain boundaries, which contributes to the deviation from ideal relaxation (β_HN_ < 1). At the same time, both ϵ_∞_ and Δϵ increase with the increase in Co^2+^ content, indicative of higher instantaneous polarizability and relaxation strength.^39^ The behavior can be rationalized by considering the ionic size effect. The larger octahedral ionic radius of Co^2+^ compared to Ni^2+^ ions favors local variations and a soft lengthening of the M-O bond distances when Co^2+^ populates the M(2) site. The slightly longer and softer M-O bonds with Co^2+^ fraction increasing are more easily polarizable under the action of an applied external electric field, which enhances the instantaneous dielectric response ϵ_∞_ as well as the overall relaxation strength Δϵ. As a consequence, the lattice contributes strongly to the fast and gradual polarization, resulting in higher ϵ_∞_ and Δϵ, together with a reduced β_*HN*_ when the Co^2+^ fraction increases.

To conclude the dielectric analysis, the KCo_1–*x*_Ni_*x*_Cr_2_(PO_4_)_3_ compounds exhibit a set of highly promising features, such as the progressive increase of ϵ′ and Δϵ with increasing the Co^2+^ content, for supercapacitor applications. A high relaxation strength Δϵ is advantageous for such devices, with ϵ_S_ ≫ ϵ_∞_.^40^ Simultaneously, higher permittivity reflects stronger intrinsic polarization, leading to faster polarization channels that facilitate high-rate operation and fast charge storage.^41^ The decrease in the β_*HN*_ parameter, indicative of an extended distribution of relaxation times, together with a significant increase in permittivity upon substitution of Ni^2+^ with Co^2+^ ions, denotes improved charge-storage capacity and high polarizability. These combined characteristics suggest the possibility of achieving both high energy and power density, as well as favorable charging/discharging kinetics. Thus, the KCo_1–*x*_Ni_*x*_Cr_2_(PO_4_)_3_ phosphate ceramics stand out as an excellent candidate for supercapacitors’ active materials.

### Equivalent Circuit and Conduction Mechanism Analysis

Deconvolution of the dielectric response allows for to separate of the contributions originating from the grains (bulk) and grain boundaries. The insights from the Havriliak–Negami analysis, particularly the deviation from ideal Debye behavior (β_*HN*_ < 1), justify using a *CPE* rather than an ideal capacitor to model the grain boundary response. The proposed equivalent circuit model used to analyze the impedance data is shown in Figure 11a. It consists of a series of resistances (R_s_) attributed to the contacts/leads, connected in series with two parallel *R*_*g*_/*C*_*g*_ - *R*_*gb*_/*CPE*_*gb*_ elements. The first element (*R*_*g*_/*C*_*g*_) models the grain response at high frequencies, while the second one (*R*_*gb*_/*CPE*_*gb*_) accounts for the more resistive grain boundary response at lower frequencies.

**Figure 11:**
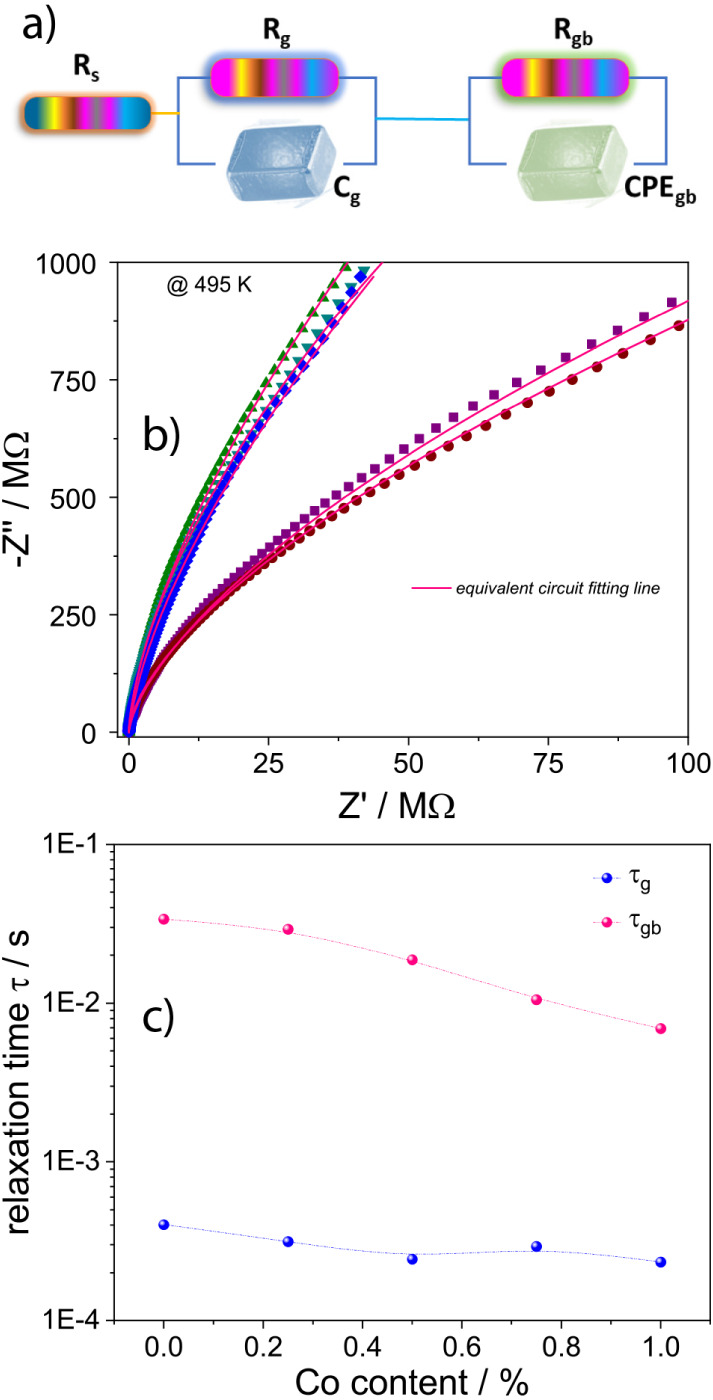
Impedance spectroscopy analysis for KCo_1–*x*_Ni*_x_* Cr_2_(PO_4_)_3_ phosphates at 495 K: a) the equivalent circuit model used for fitting, b) the Nyquist plots -Z” vs Z’; the solid red lines are the corresponding model fits, and c) compositional dependence of the relaxation times for τ*_g_* and τ*_gb_* extracted from the fits.

Figure 11b shows the complex impedance plots for all compositions at 495 K. The two overlapping semicircles in the *Z*” vs *Z*’ plots confirm the presence of two primary relaxation processes corresponding to the grain and grain boundary contributions. The solid lines represent the fits obtained using the described equivalent-circuit model, validating its applicability to the present phosphate ceramics. The relaxation times for the grains (τ_*g*_) and grain boundaries (τ_*gb*_) extracted from the resulting fits are presented as a function of the Co content in Figure 11c.

The grain boundary relaxation is 2 orders of magnitude slower than the grain relaxation (*τ*_*gb*_ ≫ τ_*g*_), due to the higher energy barrier for charge carriers to cross the boundaries. Both τ_*g*_ and τ_*gb*_ relaxation times show a monotonic decrease with Co content increasing, which means that Ni^2+^/Co^2+^ substitution facilitates faster charge transport processes, both within the grains and across their interfaces.

The charge transport mechanism in disordered materials is inherently frequency-dependent.^42^ To deepen the understanding of their behavior, the frequency dependence of the total a.c. conductivity, σ_ac_(ν), was analyzed. The conductivity spectra of the KCo_1–*x*_Ni_*x*_Cr_2_(PO_4_)_3_ phosphates exhibit distinct frequency regimes: a frequency-independent plateau corresponding to the d.c. conductivity σ_dc_, which originates from the long-range migration of charge carriers across the material at very low frequencies (ν → 0); and a dispersive regime as the frequency increases, where conductivity shows a power-law dependence specific to more localized, short-range hopping dynamics. This dual behavior is quantitatively described by Jonscher’s universal dielectric response power law:^43^

2 $$\sigma_{\mathrm{ac}}\left(\nu \right)=\sigma_{\mathrm{dc}}+A_{1}f^{s}+A_{2}f^{p}$$

where σ_dc_ is the frequency-independent conductivity, *A*_1_ and *A*_2_ are pre-exponential factors representing the relative strengths of the two frequency-dependent conduction processes, the *s* exponent (0 < *s* < 1) describes the degree of correlation between hopping charge carriers and their environment, and the *p* exponent (1 < *p* < 2) is ascribed for the localized, nontranslational motion of charge carriers. The temperature dependence of the d.c. conductivity allows us to quantify the energy barrier for the conduction process, according to the Arrhenius relationship:^44^

3 $$\sigma_{\mathrm{dc}}\cdot T=\sigma_{0}\cdot e^{-\frac{E_{a}}{k_{B}T}}$$

where σ_0_ is the pre-exponential factor, *K*_*B*_ is the Boltzmann constant, and *E*_*a*_ is the activation energy for the charge transport.

The a.c. conductivity spectra with σ_ac_ increasing as a function of frequency were analyzed using eq 2 (Figure 12a) and the compositional dependence of the extracted Jonscher exponents is plotted in Figure 12b. A true frequency-independent plateau is not observed below 1 kHz, likely due to electrode polarization effects that cause a slight decrease at the lowest frequencies. The data suggest a quasi-plateau region before entering a strongly dispersive regime above 10 kHz. The overall conductivity systematically increases with the cobalt content; the Co0Ni1 sample is the most resistive (σ_dc_ = 10^–9^ S/m), while the Co1Ni0 is the most conductive (σ_dc_ = 10^–8^ S/m) with 1 order of magnitude difference. At high frequencies, the a.c. conductivity isotherms converge toward 10^–5^ S/m for all the compositions. In addition, the slope of the conductivity in the high-frequency dispersive region becomes steeper with increasing Co content. The primary exponent *s* value lies in the range of 0.4–0.6, typical of a charge transport mechanism based on correlated, translational hopping in ion-conducting compounds.^45^ Its value shows a slight convex dependence on the Co content, with a minimum at x = 0.5, suggesting subtle changes in interionic interactions and the dimensionality of hopping pathways. The value of the second exponent, *p*, ranged between 1.6 and 1.9, exhibits a nonmonotonic increase with Co increasing, reaching a maximum for the Co0.75Ni0.25 composition. This indicates that, while the fundamental translational hopping mechanism remains consistent, with relatively close values of the *s* exponent across all samples, the dynamics of the localized short-range hopping become more pronounced and frequency-dependent, and are significantly influenced by the Ni^2+^/Co^2+^ ratio. The increase in *p* with Co increasing directly corresponds to the steeper slope of the conductivity spectra observed in Figure 12a, confirming that localized dynamics become a more dominant contribution to the overall a.c. conductivity at high frequencies.

**Figure 12:**
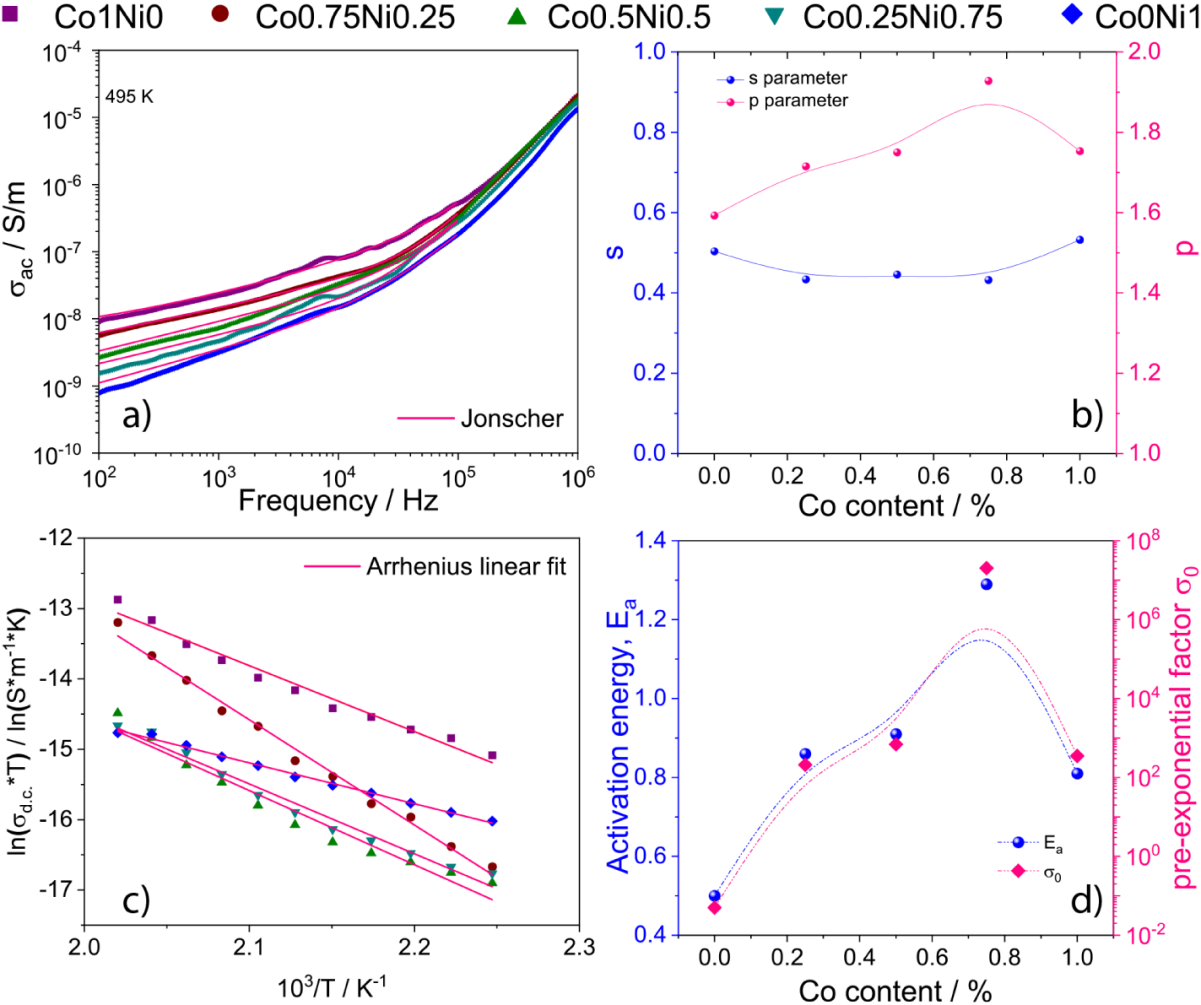
Analysis of the charge transport mechanism in KCo_1–*x*_Ni*_x_* Cr_2_(PO_4_)_3_ phosphates: (a) frequency dependence of a.c. conductivity at 495 K; solid lines are the results of the fit using eq 2, (b) compositional dependence of the Jonscher law exponents, *s* and *p*, (c) Arrhenius plots of ln­(σ_dc_ · *T*) vs 10^3^/*T* used to determine the activation energy for d.c. conduction, (d) evolution of the activation energy *E_a_*, and the pre-exponential factor σ_0_ as a function of the Co content.

The activation energy *E*_*a*_ of the charge transport was determined from the slope of the Arrhenius plots (Figure 12c), and its compositional dependence is shown in Figure 12d. The intensified hopping dynamics in the Co compounds strongly suggest a reduced energetic barrier to charge transport. Surprisingly, the *E*_*a*_ results reveal a counterintuitive trend. Instead of decreasing, the activation energy systematically increases with Co content: from 0.5 eV for Co0Ni1 to 0.86 eV for Co0.25Ni0.75, 0.91 for Co0.5Ni0.5, reaching a maximum of 1.29 eV for Co0.75Ni0.25, before decreasing again to 0.81 eV for Co1Ni0. This is in apparent contradiction with the conductivity spectra (Figure 12a), where a higher Co content leads to a higher overall a.c. conductivity. The conductivity increasing despite a rising energy barrier can only be explained by considering the role of the pre-exponential factor σ_0_ in eq 3. A sharp increase in the pre-exponential factor σ_0_ would completely overwhelm the opposing exponential effect of the higher activation energy, and thus the observed behavior is physically possible. To verify this hypothesis, σ_0_ was calculated from the y-intercept of each Arrhenius fit, and the results are presented in Figure 12d. σ_0_ increases by more than 8 orders of magnitude, with a maximum at the Co0.75Ni0.25 composition where *E*_*a*_ is maximal. Although Co^2+^ substitution increases the energy barrier for individual charge carrier jumps, this effect is more than compensated for by a sharply increase in the concentration of mobile charge carriers. This is a manifestation of the Meyer-Neldel (MN) compensation rule that leads to higher total conductivity.^46^ Thus, the Ni^2+^/Co^2+^ substitution has a dual effect: (i) it increases the hopping energy barrier for individual carriers, and (ii) it simultaneously liberates a massive number of previously immobile charge carriers. This pronounced increase in the concentration of mobile carriers is the dominant factor responsible for the overall enhancement of the a.c. conductivity.

The strategy of substituting Ni^2+^ ions with Co^2+^ ions in KCo_1–*x*_Ni_*x*_Cr_2_(PO_4_)_3_ phosphates successfully produces a material with a particularly combination of properties for applications in supercapacitor devices. The improvements include higher charge-storage capacity, as evidenced by increased dielectric permittivity and relaxation strength, and enhanced power capability due to higher overall conductivity.

### Energy Storage Potential

The energy storage capability of the synthesized samples was measured by fabricating all-in-one supercapacitor devices using a mixture of graphite and the developed Ni/Co phosphates as the electrode material and 1 M KOH aqueous solution as the electrolyte. The supercapacitor devices used glass fiber as a separator and were measured in a two-electrode system.

Cyclic voltammetry (CV) is a widely used electrochemical technique for evaluating supercapacitor performance, as it provides insights into reaction kinetics, charge storage mechanisms, and current responses. Figure 13a-e shows the CV curves of all assembled SC devices recorded at different scan rates, while the inset of Figure 13f compares the CVs measured at 100 mV/s. The ideal electric double-layer capacitor (EDLC)-type behavior for carbon-based materials is characterized by nearly rectangular CVs, reflecting a constant-current response over the applied voltage range,^47^ whereas metal oxides are generally associated with pseudocapacitance due to a higher tendency of metal ions to facilitate faradic reactions. As shown in Figure 13a-e, the CV shape evolves systematically with Ni/Co ratio: increasing the Co content shifts the profiles closer to the ideal EDLC form. The current response also increases with higher Co^2+^ concentration, while the Co0.5Ni0.5, Co0.75Ni0.25, and Co1Ni0 compounds based SCs show similarly high maximum currents, though with differences in CV area. In contrast, the Co0Ni1-based SC device produces distorted, resistive-type CVs that deviate from the ideal rectangular shape.^48^ These curves exhibit lower current responses and smaller enclosed areas, directly correlating with reduced specific capacitance. Although the cyclic voltammograms exhibit a predominantly rectangular shape characteristic of electric double-layer capacitance, the devices also exhibit pseudocapacitance, as Co can exist in different oxidation states and facilitate redox reactions. The devices with higher Co content, such as Co1Ni0, Co0.75Ni0.25, and Co0.5Ni0.5, exhibit a sudden increase in current at the maximum voltage, indicative of redox activity. The absence of sharp redox peaks and the preservation of a capacitive CV profile indicate that these Faradaic reactions are fast, reversible, and confined to the electrode–electrolyte interface. Such surface-controlled redox processes result in a pseudocapacitive response, with kinetics sufficiently rapid to mimic ideal capacitive behavior.^49^ The electrochemical response can therefore be classified as surface-controlled pseudocapacitance. The micrometer-sized particles of the synthesized material also play a critical role in limiting the pseudocapacitive contribution in the tested devices, thereby affecting the overall device performance.

**Figure 13:**
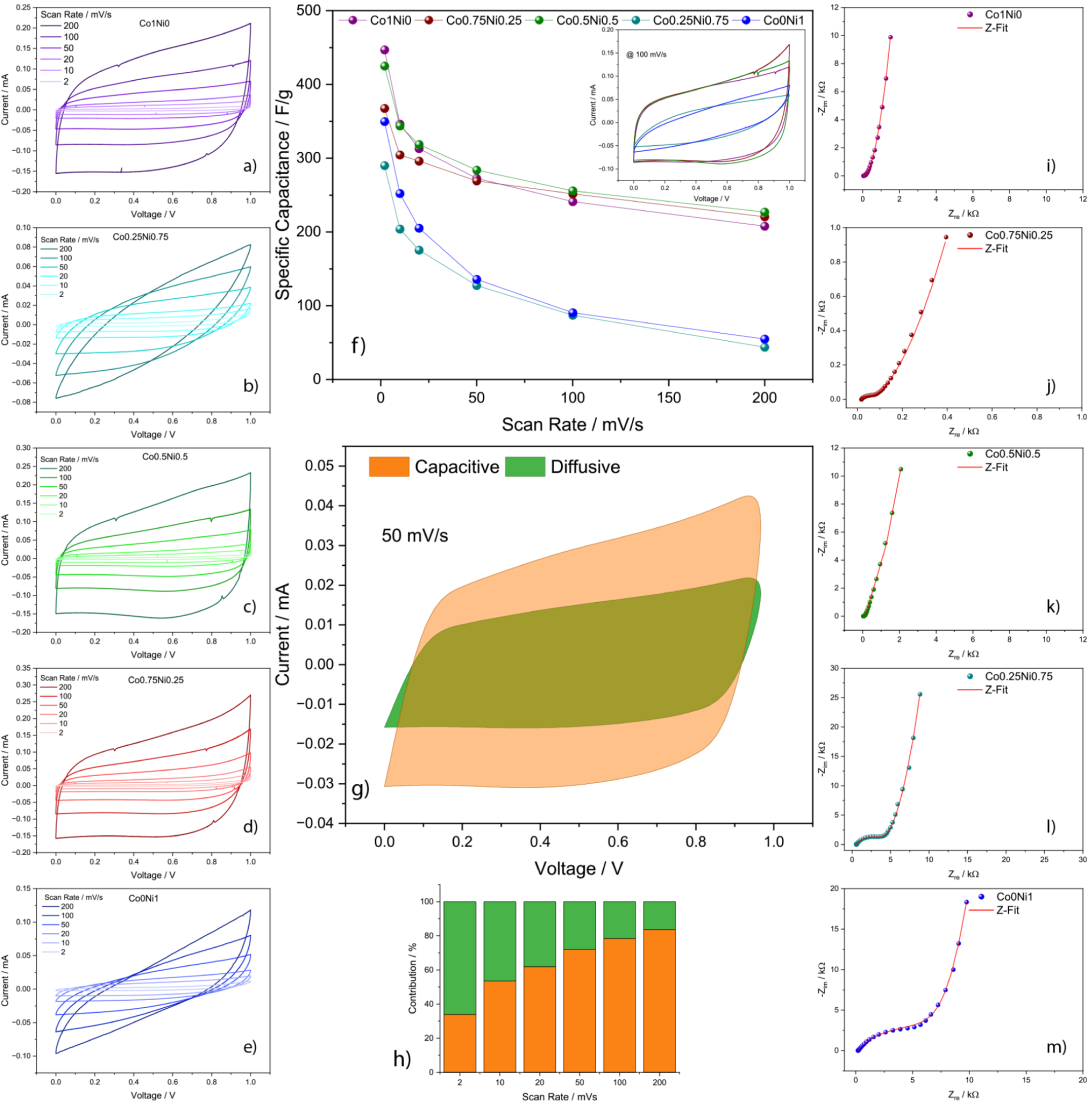
CV measurements of the SC devices based on Co1Ni0 (a), Co0.75Ni0.25 (b), Co0.5Ni0.5 (c), Co0.25Ni0.75 (d), and CoNi1 (e) perfomed at different scan rates. C*_s_* values of all KCo_1–*x*_Ni*_x_* Cr_2_(PO_4_)_3_ compounds at different scan rates (f), derived from the CV measurements. The inset of (f) compares the CV curves of all compounds at 100 mV/s. Capacitive and diffusive contributions to the energy storage mechanisms presented on the CV curve (g) measured at 50 mV/S of the SC device employing Co1Ni0, and as a function of the scan rate (h). Nquist plot together with the equivalent circuit fit results of the graphite-based SC devices using Co1Ni0 (i), Co0.75Ni0.25 (j), Co0.5Ni0.5 (k), Co0.25Ni0.75 (l), and CoNi1 (m).

Strong Cr^3+^-Co^2+^ exchange interactions, as reported in the EPR section, directly affect supercapacitor performance with increasing Co^2+^ content. Strong electronic coupling, which facilitates rapid electron transfer between redox-active centers and promotes electronic delocalization, is favorable for fast, reversible faradaic reactions, thereby improving supercapacitor performance.^50,51^ However, larger particle sizes hinder charge transport by restricting ion diffusion and reducing the specific surface area. Consequently, the number of electrochemically active sites available for faradaic reactions is limited, thereby suppressing the device’s pseudocapacitance.

The specific capacitance of the devices was calculated from CV measurements using the following equation: $C_{s}=\frac{\int_{V_{1}}^{V_{2}}I\left(V\right)dV}{2mK\Delta V}$ , where *C*_*s*_ is the specific capacitance, $\int_{V_{1}}^{V_{2}}I\left(V\right)dV$ represents the area under the CV curve, *m* is the mass of the active electrode material, *K* is the scan rate at which the CV was acquired and Δ*V* is the voltage window.^52^ Figure 13f shows the specific capacitance of all the SC devices plotted as a function of the scan rate. The Co0Ni1 and Co0.25Ni0.75 compound-based SC devices showed the lowest specific capacitance values at each scan rate, as expected, based on the CV curves and previously reported dielectric properties. The devices with increased Co content exhibited very similar specific capacitance values, with the Co1Ni0-based SC device achieving the highest specific capacitance at 2 mV/s, approximately 446 F/g, followed by the Co0.5Ni0.5-based device at 425 F/g. The trend in the supercapacitor device’s performance indicates a positive impact of Co ions, which facilitate charge transfer, as observed in previously published supercapacitor results.^53^

The Dunn analysis of the Co1Ni0-based SC device was performed to determine the contributions of capacitive and diffusive charge-storage mechanisms. The equation used to express the different contributions to the energy storage mechanism is described as $I\left(V\right)=K_{1}\cdot v+K_{2}\cdot \sqrt{v}$ , where *K*_1_ · *v* represents the capacitive current, $K_{2}\cdot \sqrt{v}$ represents the diffusive current, and *v* is the scan rate. The relative contributions of each mechanism can be quantified by analyzing the slope and intercept of the Dunn plot, which represent the *K*_1_ and *K*_2_ constants.^54^ Figure 13h shows the proportional contributions plotted as a function of the scan rate. As shown in Figure 13h, the capacitive mechanism dominates across all scan rates, with only a small contribution from diffusive processes at lower scan rates. This complements our CV observation, in which we report that higher charge-storage dominance in EDLCs arises from their carbon content, whereas a small faradic contribution arises from Co ions in the phosphates. This confirms that the Co1Ni0-based SC device stores charge through a combination of the electric double-layer mechanism and pseudocapacitive contributions. These results are consistent with the CV and GCD analyses, indicating increased EDLC-type behavior in the Co-rich compounds. Figure 13g shows the Dunn analysis in CV representation at 50 mV/s, and we observe that the major part of the charge storage process is due to the capacitive behavior.

The b-value for all SC devices is calculated by plotting log­(*i*) against log­(*v*), where *v* is the scan rate. The slope of this plot corresponds to the b-value, which indicates the charge-storage mechanism. A b-value near 0.5 shows diffusion-controlled behavior, while a b-value near 1 shows capacitive-controlled behavior. The b-value plot of each device is presented in Figure S4, while the resulting b-value after linear fitting is shown in Table 3. The values show that the electrochemical response is predominantly surface-controlled, with a minor diffusion contribution, particularly in higher-Co-containing phosphates-based SC devices, which are the best performers, indicating that the charge-storage mechanism is dominated by double-layer capacitance from graphite and a surface-confined faradic reaction from the CoNi-based materials.

**Table 3:** Equivalent Circuit Values Obtained from the Z-Fit of the Nyquist Data and Ragone Plot Values for the KCo_1–*x*_Ni*_x_* Cr_2_(PO_4_)_3_ Based SCs Devices

Compound	b-value	R_s_/Ω	R_ct_/Ω	*C*_*dl*_/μF	*E*_*D*_/Wh/kg	*P*_*D*_/kW/kg
Co0Ni1	0.64	190	7112	64	35	1272
Co0.25Ni0.75	0.7	546	3548	20.24	28.19	1018
Co0.5Ni0.5	0.86	25.6	112	347	47.63	1732
Co0.75Ni0.25	0.86	17.84	58.4	35.85	42.28	1537
Co1Ni0	0.83	39.65	76.7	78.2	48.18	1752

Figure 13i-m presents the Nyquist plots of all devices, with the imaginary part plotted against the real part of the impedance. Consistent with the CV results, SC devices based on compounds with higher Co content exhibit a distinct Nyquist response compared with Ni-dominated systems. The x-intercept in the high-frequency region corresponds to the series resistance R_s_, representing the electrochemical cell’s total ohmic resistance, including contributions from the electrolyte, electrodes, and contacts.^55^ As shown in the Figure 13i-m, the Co0Ni1 and Co0.25Ni0.75 devices exhibit relatively high R_s_ values, while devices with higher Co content display lower R_s_. The semicircle observed in the high-frequency region is associated with charge-transfer resistance R_ct_;^55^ a larger semicircle diameter is evident for Ni-rich electrolytes, indicating higher R_ct_ values. At low frequencies, the Nyquist plots approach a near-vertical line, characteristic of capacitive ion storage. The most vertical responses are observed for Co0.5Ni0.5, Co0.75Ni0.25, and Co1Ni0, confirming their EDLC-dominated charge storage with efficient ion diffusion. In contrast, Ni-rich systems exhibit a noticeable tilt away from vertical, reflecting diffusion limitations. The values of the physical parameters were obtained by fitting the Nyquist plots with an equivalent circuit. The values obtained are shown in the Table 3. In general, the Co-enriched electrolyte devices exhibited lower R_s_ and R_ct_ and higher *C*_*dl*_, whereas the Ni-enriched electrolyte devices exhibited higher resistance values, which explains the performance difference between the SC devices.

Figure 14a presents the charge–discharge curves of all devices measured at 1 A/g, plotting voltage against time. The Co0.5Ni0.5-based SC device exhibits the longest discharge time, closely followed by Co1Ni0. This trend is consistent with the earlier electrochemical results: Ni-rich electrolytes yield shorter discharge times, whereas increasing Co content improves device performance. The charge–discharge profiles display the almost triangular shape characteristic of EDLC behavior, indicating symmetrical charging and discharging.^56^ This effect is particularly pronounced for the Co-rich compounds, consistent with the CV results. The pseudocapacitance behavior is also not visible in the GCD curves due to surface-confined, fast faradic reactions, as explained in the CV section. Figure 14b shows the cyclic stability of all SC devices, plotting capacitance retention as a function of the charge–discharge cycle number. All devices generally showed very good cyclic stability after 5000 cycles. Almost all devices exhibit increased capacitance retention with increasing cycle number. This phenomenon is unusual but not new for supercapacitor devices. This improvement can be attributed to progressive activation of the electrode–electrolyte interface, with repeated cycling enhancing ion accessibility and improving electrode wetting. Additionally, the continuous redistribution of ions within the porous structure and the possible activation of previously inaccessible sites may contribute to the gradual increase in capacitance. This phenomenon has also been reported for supercapacitor devices, which explains the increase in capacitance observed for carbon-based and other electrodes.^57–59^

**Figure 14:**
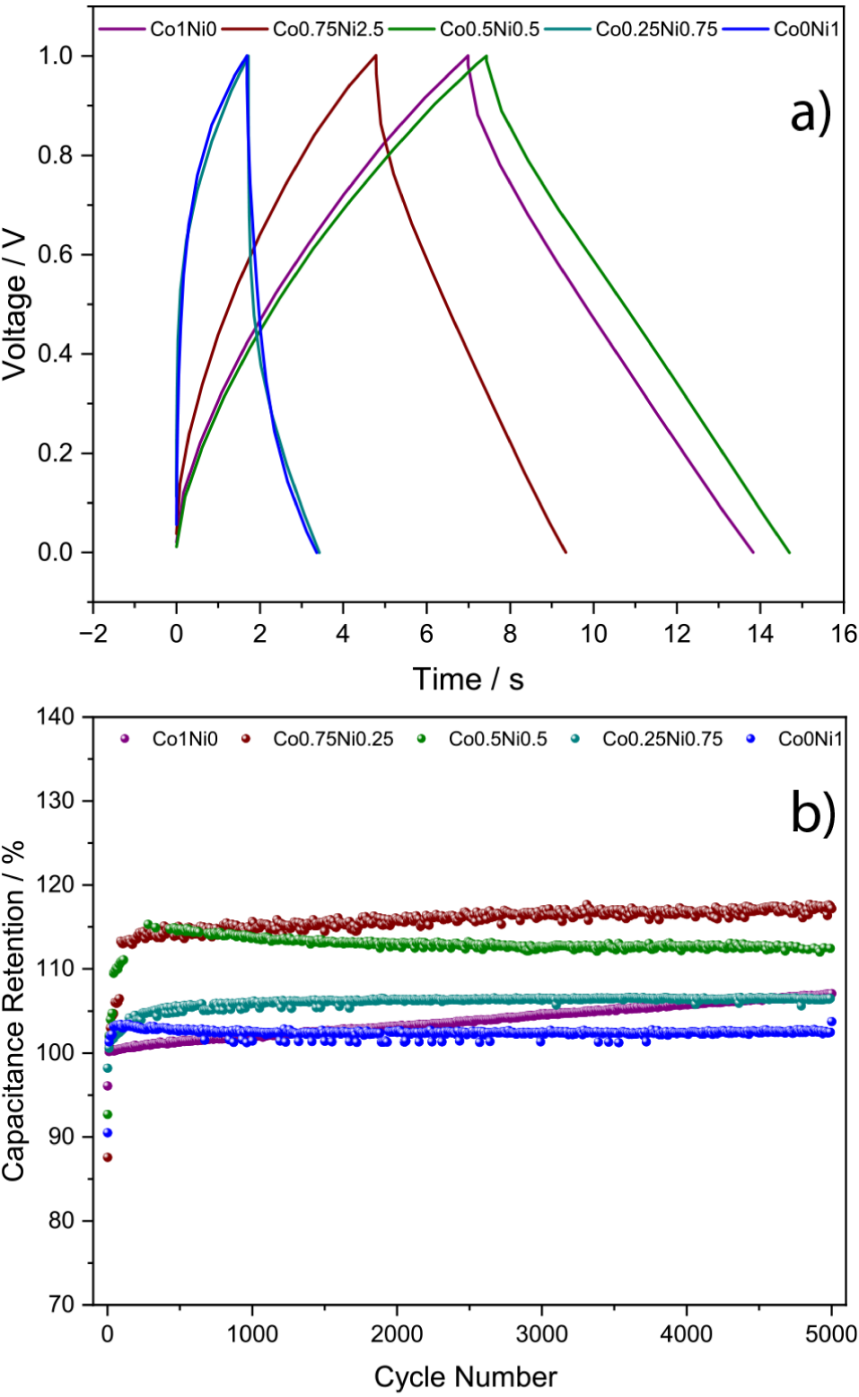
Galvanostatic Charge–Discharge (a) and Capacitance retention against cycle number (b) for KCo_1–*x*_Ni*_x_* Cr_2_(PO_4_)_3_ based SCs devices.

Table 3 shows the energy and power density values of all the SC devices calculated from each device’s highest specific capacitance values. Energy and power density values are the standard comparison metrics for energy storage devices, commonly known as the Ragone plot.^60,61^ In general, increased Co content in devices led to improved energy and power densities. The Co1Ni0 and Co0.5Ni0.5-based SC devices outperform the other devices, indicating that an optimal CoNi ratio improves overall device performance. Both Co1Ni0 and Co0.5Ni0.5 exhibited competitive performance, with the Co1Ni0 device slightly outperforming in specific capacitance, energy density, and power density. In the GCD measurements, however, the Co0.5Ni0.5 device exhibited a slightly shorter discharge time than the Co1Ni0. To further highlight the effect of the Co–Ni-containing phosphates on energy storage properties, Figure S3 shows electrochemical measurements using pure graphite in a symmetric configuration in 1 M KOH. The results show that although the graphite-based SC device resembles the highest specific capacitance among the SC devices presented in this study, it exhibited a sharp drop in cyclic stability, with retention dropping to 75% after 5000 cycles. However, the addition of CoNi-based phosphates tends to improve the device’s cyclic stability, as shown in Figure 14b, and only a slight reduction in specific capacitance was observed compared to the graphite-based SC device.

SEM analysis indicates that the synthesized particles are predominantly in the micrometer range. In conventional supercapacitor materials, such morphology may limit ion diffusion kinetics due to increased transport path length and reduced external surface area. However, the present compounds crystallize in the α-CrPO_4_ structural type (space group *Imma*), characterized by a robust three-dimensional framework composed of corner-sharing MO_6_ (M = Co, Ni, Cr) octahedra and PO_4_ tetrahedra. This arrangement generates a three-dimensionally interconnected tunnel system in which K^+^ ions are accommodated within structural cavities. The presence of such intrinsic channels provides bulk ionic diffusion pathways that are not solely dependent on external particle size. Consequently, ion transport and redox activity are governed not only by surface effects but also by the crystallographic framework topology. The relatively high specific capacitance of 447 F/g demonstrates effective electrochemical accessibility of the transition-metal redox centers despite the micrometric morphology. These results suggest that charge storage involves diffusion-assisted faradaic processes within the framework. Further enhancement of rate capability could be achieved through particle downsizing or hierarchical porosity engineering.

The supercapacitor results are consistent with the permittivity measurements, which indicate that increasing Co content enhances polarizability and dielectric permittivity. A higher permittivity can facilitate more effective ion polarization at the electrode–electrolyte interface, thereby promoting improved charge separation and accumulation within the electric double layer.^62^ Substituting Ni^2+^ with the larger Co^2+^ ions induces a slight lattice expansion and reduces the polarizing power at the M(2) site, yielding softer, more easily deformable M(2)-O bonds with higher instantaneous polarizability under an electric field, directly reflected in the increased high-frequency permittivity ϵ^∞^, dielectric permittivity ϵ′, and relaxation strength Δϵ identified in the Havriliak–Negami analysis. A highly polarizable bulk creates a stronger local internal field that more effectively screens electrostatic repulsions between solvated ions, facilitating denser ion accumulation and improved separation at the interface, thereby promoting the formation of the electric double layer. This aligns with Dunn’s analysis, which found that charge storage is mainly capacitive. A higher dielectric permittivity ϵ′ in the electrode material enables more effective ion polarization at the electrode–electrolyte interface, promoting superior charge separation and accumulation within the electric double layer. This is the primary mechanism supporting the high specific capacitance of 447 F/g recorded for the Co1Ni0 composition. Furthermore, the increase in permittivity and the distribution of relaxation times, as evidenced by the β_NM_ parameter, indicate the formation of faster polarization channels that support high-rate operations and rapid charge–discharge kinetics, effectively translating the material’s intrinsic dielectric agility into a superior power density of 1752 W/kg. The dielectric flexibility, combined with a significant rise in mobile charge-carrier concentration, consistent with the Meyer-Neldel rule, results in a notably lower charge-transfer resistance (R_ct_) in the impedance spectra of Co-rich samples, enabling fast, surface-confined faradaic reactions that resemble ideal capacitive behavior.

Compared with the already published state of the art for SC devices, the Ragone plot values for graphite electrodes showed an encouraging trend for devices based on graphite in combination with the phosphate compounds tested in this work.^63^ reported using a biochar-modified graphite electrode for supercapacitor applications with 1 M KCl as the electrolyte. They reported an energy density of 21.62 Wh/kg and a power density of 400 W/kg for the modified graphite electrode, which are lower than those reported in this work. Similarly,^64^ demonstrated the use of polypyrrole microtubes on a disposable pencil graphite electrode in an SC application, using 0.1 M KClO_4_ and 0.5 M H_2_SO_4_ as electrolytes to test the electrode material’s electrochemical performance. The maximum values reported in this study were 10 Wh/kg for energy density and 1500 W/kg for power density. The comparison shows the positive impact of phosphate compounds on the performance of carbon-based SC devices.

## Conclusions

The KCo_1–*x*_Ni_*x*_Cr_2_(PO_4_)_3_ phosphates (x = 0, 0.25, 0.5, 0.75, 1) were synthesized via a solid-state reaction and were found to crystallize in the orthorhombic system, adopting the *Imma* space group, isotypic with α-CrPO_4_. Spectroscopic IR measurements (Raman and FT-IR) confirmed the presence of characteristic P–O and M-O vibrational modes, consistent with the existence of a single PO_4_ group. EDX and SEM analyses verified the chemical composition and morphology, confirming the formation and aggregation state of the synthesized materials. EPR spectroscopy measurements revealed the presence of paramagnetic Cr^3+^ ions, with the EPR signal intensity increasing progressively as the Co content increased in the mixed compositions. These findings highlight the structural stability of the phosphate framework across the range of substitutions and underscore the system’s magnetic tunability via controlled cation substitution.

The dielectric and charge transport properties of the KCo_1–*x*_Ni_*x*_ solid solution were systematically investigated to assess the strategic effect of Ni^2+^/Co^2+^ substitution. The materials were found to exhibit a stable, intrinsically paraelectric response. The analysis demonstrated that compositional tuning is a highly effective method for controlling the material’s properties, with cobalt substitution systematically increasing the overall polarizability and dielectric permittivity while maintaining low dielectric loss. The electrochemical performance of the KCo_1–*x*_Ni_*x*_Cr_2_(PO_4_)_3_ phosphates was evaluated in graphite-based supercapacitor devices. The results suggest that the device’s electrochemical performance improves with increasing cobalt substitution in the material. The CV shape approached that of an ideal EDLC, and an increased current response was observed upon Co^2+^ substitution in the phosphates, resulting in higher specific capacitance values. Electrochemical impedance spectroscopy results indicate lower R_s_ and R_ct_ values as the Co-ion content increases in SC devices. While charge–discharge curves show a longer discharge time. Increasing the Co content improves the energy storage performance, with a maximal specific capacitance of 447 F/g, and energy and power densities of 48.18 Wh/kg and 1752 W/kg, respectively.

A detailed investigation of the charge-transport mechanism revealed a complex, counterintuitive phenomenon. Contrary to the behavior of a simple thermally activated conductor, the overall conductivity increased with cobalt content despite a simultaneous and significant increase in the activation energy for charge transport. The compensation law quantitatively explains this apparent paradox. The detrimental effect of the higher energy barrier is overcome by a multiorder-of-magnitude increase in the pre-exponential factor, indicating that Co substitution sharply increases the concentration of mobile charge carriers. This unique mechanism, where a massive increase in active charge carriers drives high conductivity, is particularly advantageous for energy storage.

The KCo_1–*x*_Ni_*x*_Cr_2_(PO_4_)_3_ phosphates are highly promising supercapacitor electrode materials, combining high charge-storage capacity, derived from their enhanced permittivity, with improved power capability, enabled by their higher overall conductivity. These results validate Ni^2+^/Co^2+^ substitution as a compelling strategy for designing advanced electrode materials.

## Supplementary Material



## Notes

XRD refinement, EDX emission spectra, tables with crystallographic data for the KCo_1–*x*_Ni_*x*_Cr_2_(PO_4_)_3_ phosphates, b-value plots, and graphite-based SC device evaluation (PDF)

## #

Z.M. and R.E.P. Contributed equally
