# Supplementary material for: Compositional Tuning of Mixed Chromium Monopotassium Phosphates by Ni2+/Co2+ Substitution for Energy Storage Applications
Source: Inorg Chem. 2026 May 28;65(22):12107–25. doi: 10.1021/acs.inorgchem.5c05545 (PMC13250973; doi:10.1021/acs.inorgchem.5c05545)
Supplement: Supplementary file 1 [file ic5c05545_si_001.pdf]

Supplementary information file:

Compositional tuning of mixed chromium monopotassium phosphates by Ni^{2+}/Co^{2+} substitution for energy storage applications

Zaineb Mighri,[†,‡,¶] Roxana Elena Patru,[†,‡] Ameen Uddin Ammar,[§] Lucia Nicoleta Leonat,[‡] Habib Nasri,[¶] Aurelian Catalin Galca,[*,‡,||] and Arpad Mihai Rostas[*,§]

† Contributed equally

‡ Laboratory of Complex Heterostructures and Multifunctional Materials (HeCoMat), National Institute of Materials Physics, Atomistilor 405A, 077125 Magurele, Romania

¶ Laboratory of Physical Chemistry of Materials (LR01ES19), Faculty of Sciences of Monastir, University of Monastir, Avenue de l'Environnement, 5019 Monastir, Tunisia

§ National Institute for Research and Development of Isotopic and Molecular Technologies, Donat 67-103, 400293 Cluj-Napoca, Romania

|| International Centre for Advanced Training and Research in Physics, Atomistilor 409, 077125 Magurele, Ilfov, Romania

E-mail: ac galca@infim.ro; arpad.rostas@itim-cj.ro

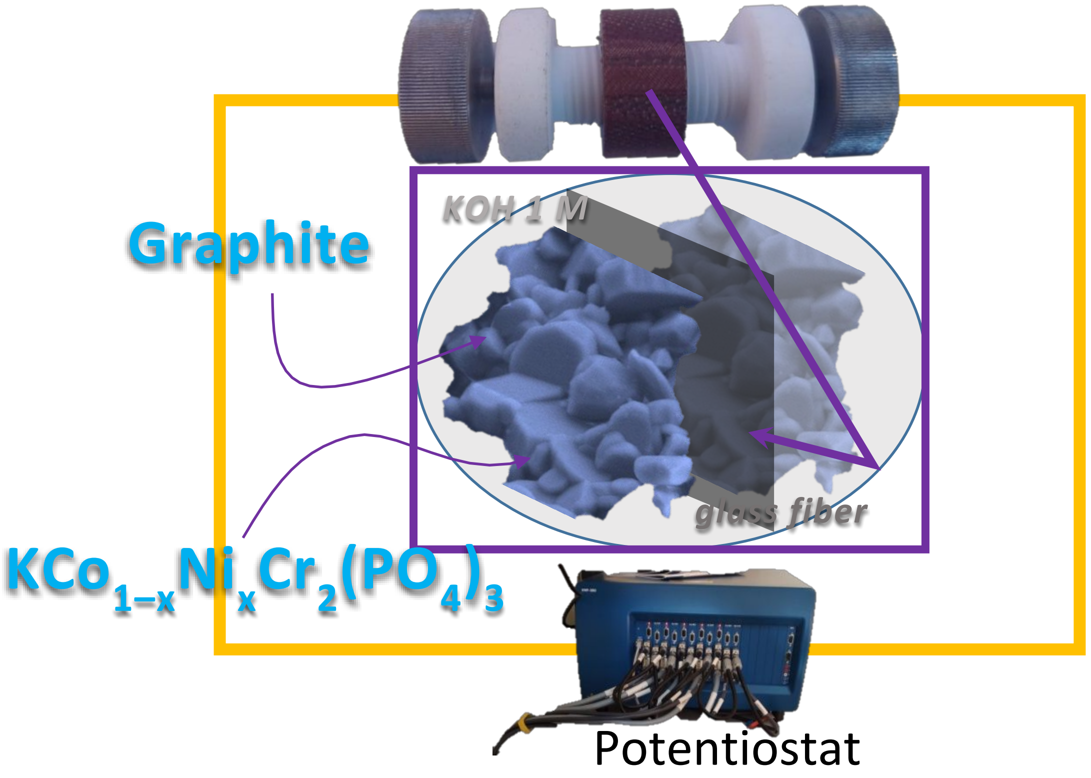

Scheme S1: Schematic representation of the supercapacitor cell used to assess the energy storage properties of the synthesized compounds.

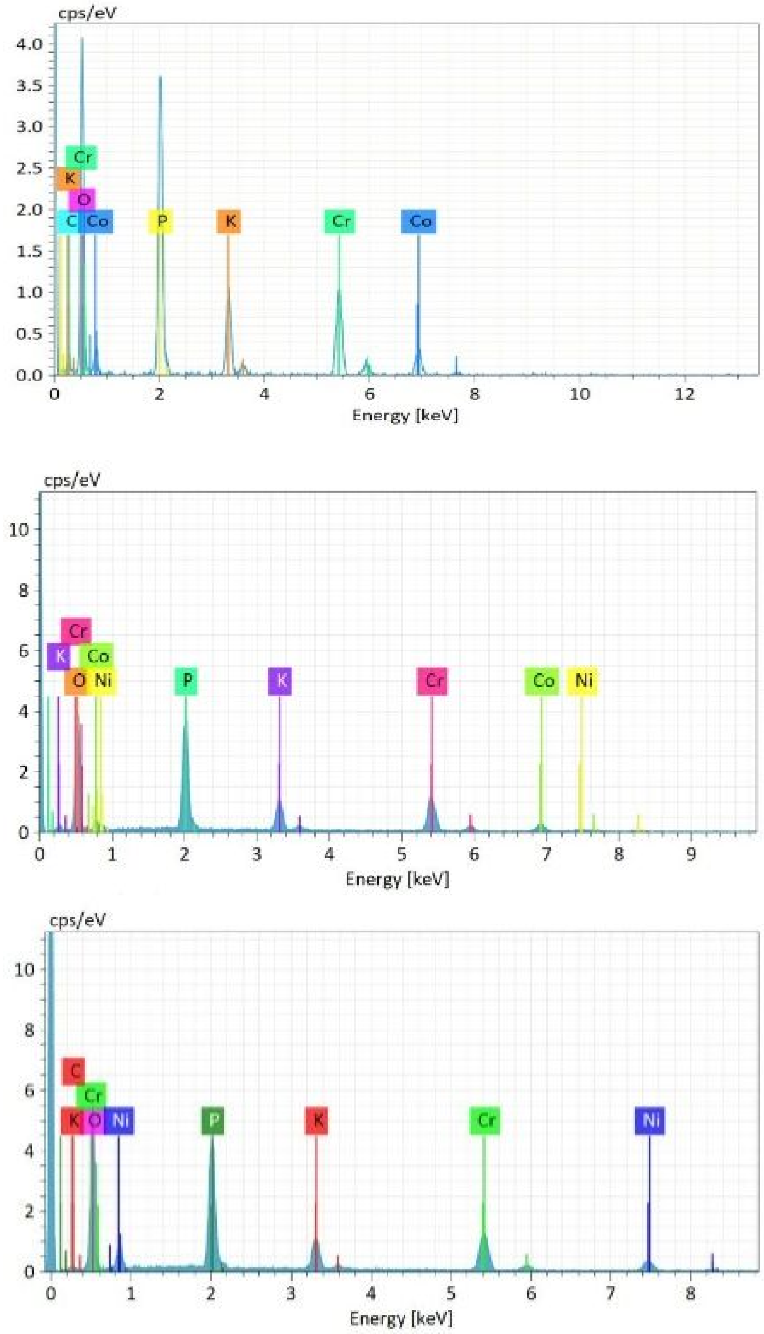

Figure S1: EDX emission spectra of the Co1Ni0, Co0.5Ni0.5, and Co0Ni1.

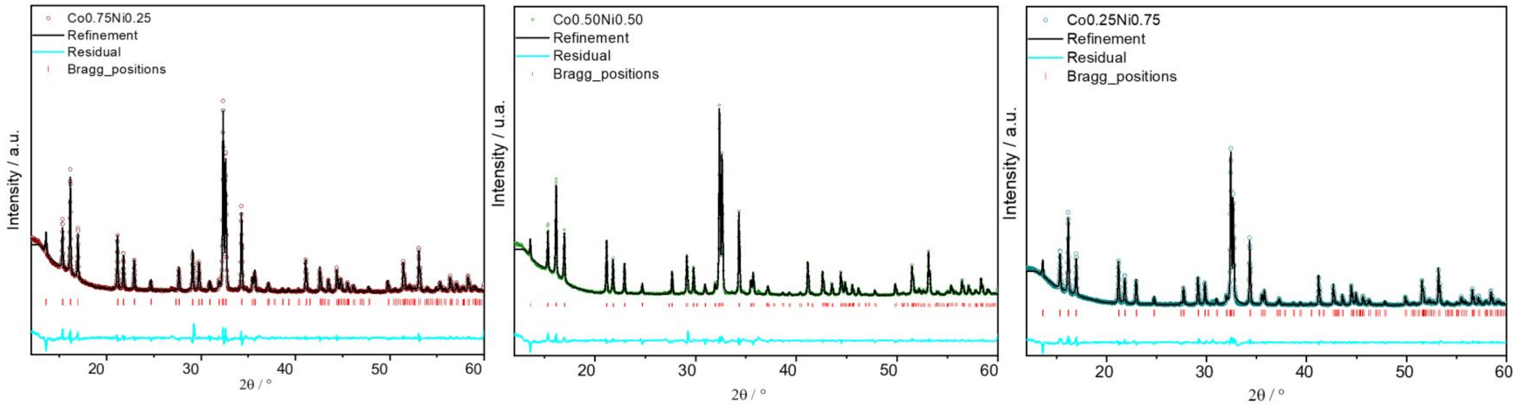

Figure S2: Rietveld analysis results of Co0.75Ni0.25, Co0.5Ni0.5, and Co0.25Ni0.75 .

Table S1: Crystallographic data and Goodness of Refinement from X-ray data for $KCo_{1-x}Ni_xCr_2(PO_4)_3$

	**Co1Ni0**	**Co0.75Ni0.25**	**Co0.50Ni0.50**	**Co0.25Ni0.75**	**Co0Ni1**
**Chemical Formula**	$KCoCr_2(PO_4)_3$	$KCo_{0.75}Ni_{0.25}Cr_2(PO_4)_3$	$KCo_{0.50}Ni_{0.50}Cr_2(PO_4)_3$	$KCo_{0.25}Ni_{0.75}Cr_2(PO_4)_3$	$KNiCr_2(PO_4)_3$
**a (Å)**	10.472(3)	10.461(3)	10.453(3)	10.449(3)	10.455(3)
**b (Å)**	13.063(3)	13.046(3)	13.033(3)	13.007(3)	13.022(3)
**c (Å)**	6.451(2)	6.451(2)	6.448(2)	6.439(3)	6.443(3)
**V(Å^3)**	882.504	880.424	878.357	875.094	877.151
**Z**	4	4	4	4	4
**R_p(%)**	16.25	17.41	14.84	15.61	15.43
**R_{wp} (%)**	11.73	13.70	11.29	12.86	11.57
**R_{exp} (%)**	4.18	3.72	3.02	3.44	3.91
**χ^2**	2.81	3.69	3.73	3.74	2.96
**R_B (%)**	1.013	1.219	0.954	1.687	1.345

Table S2: Atomic coordinates, occupation and isotropic thermal displacement parameters for $KCo_{1-x}Ni_xCr_2(PO_4)_3$ samples.

Compounds	***Site [Wickoff]***	***Point symmetry***	***Atom***	***x***	***y***	***z***	B_{iso}(Å^2)
Co1Ni0	X(1) [*4e*]	mm2	K	0	1/4	0.4036(5)	0.5(1)
Co0.75Ni0.25	X(1) [*4e*]	mm2	K	0	1/4	0.4022(5)	0.5(1)
Co0.50Ni0.50	X(1) [*4e*]	mm2	K	0	1/4	0.4045(3)	0.5(1)
Co0.25Ni0.75	X(1) [*4e*]	mm2	K	0	1/4	0.4027(3)	0.5(1)
Co0Ni1	X(1) [*4e*]	mm2	K	0	1/4	0.4048(2)	0.5(1)
Co1Ni0	M(1) [*4a*]	2/m..	Cr	0	0	0	0.5(1)
Co0.75Ni0.25	M(1) [*4a*]	2/m..	Cr	0	0	0	0.5(1)
Co0.50Ni0.50	M(1) [*4a*]	2/m..	Cr	0	0	0	0.5(1)
Co0.25Ni0.75	M(1) [*4a*]	2/m..	Cr	0	0	0	0.5(1)
Co0Ni1	M(1) [*4a*]	2/m..	Cr	0	0	0	0.5(1)
Co1Ni0	M(2) [*8g*]	.2.	0.50Cr + 0.50Co	1/4	0.6328(5)	1/4	0.5(1)
Co0.75Ni0.25	M(2) [*8g*]	.2.	0.50Cr+0.36Co + 0.14Ni	1/4	0.6340(5)	1/4	0.5(1)
Co0.50Ni0.50	M(2) [*8g*]	.2.	0.50Cr+0.25Co + 0.25Ni	1/4	0.6302(3)	1/4	0.5(1)
Co0.25Ni0.75	M(2) [*8g*]	.2.	0.50Cr+0.15Co + 0.35Ni	1/4	0.6322(5)	1/4	0.5(1)
Co0Ni1	M(2) [*8g*]	.2.	0.50Cr + 0.50Ni	1/4	0.6335(2)	1/4	0.5(1)
Co1Ni0	P(1) [*4e*]	mm2	P	0	1/4	0.9152(5)	0.5(1)
Co0.75Ni0.25	P(1) [*4e*]	mm2	P	0	1/4	0.9190(4)	0.5(1)
Co0.50Ni0.50	P(1) [*4e*]	mm2	P	0	1/4	0.9191(5)	0.5(1)
Co0.25Ni0.75	P(1) [*4e*]	mm2	P	0	1/4	0.9105(4)	0.5(1)
Co0Ni1	P(1) [*4e*]	mm2	P	0	1	0.9122(3)	0.5(1)
Co1Ni0	P(2) [*8g*]	.2.	P	1/4	0.4256(3)	1/4	0.5(1)
Co0.75Ni0.25	P(2) [*8g*]	.2.	P	1/4	0.4268(5)	1/4	0.5(1)
Co0.50Ni0.50	P(2) [*8g*]	.2.	P	1/4	0.4267(4)	1/4	0.5(1)
Co0.25Ni0.75	P(2) [*8g*]	.2.	P	1/4	0.4264(3)	1/4	0.5(1)
Co0Ni1	P(2) [*8e*]	.2.	P	1/4	0.4267(5)	1/4	0.5(1)
Co1Ni0	O(11) [*8h*]	m..	O	0	0.6508(4)	0.9532(6)	0.5(1)
Co0.75Ni0.25	O(11) [*8h*]	m..	O	0	0.6502(3)	0.9575(4)	0.5(1)
Co0.50Ni0.50	O(11) [*8h*]	m..	O	0	0.6477(5)	0.9544(3)	0.5(1)
Co0.25Ni0.75	O(11) [*8h*]	m..	O	0	0.6505(2)	0.9541(5)	0.5(1)
Co0Ni1	O(11) [*8h*]	m..	O	0	0.6518(2)	0.9527(7)	0.5(1)
Co1Ni0	O(12) [*8i*]	.m.	O	0.3799(3)	1/4	0.7291(4)	0.5(1)
Co0.75Ni0.25	O(12) [*8i*]	.m.	O	0.3804(3)	1/4	0.7328(5)	0.5(1)
Co0.50Ni0.50	O(12) [*8i*]	.m.	O	0.3832(5)	1/4	0.7315(3)	0.5(1)
Co0.25Ni0.75	O(12) [*8i*]	.m.	O	0.3812(3)	1/4	0.7337(2)	0.5(1)
Co0Ni1	O(12) [*8i*]	.m.	O	0.3801(5)	1/4	0.7350(8)	0.5(1)
Co1Ni0	O(21) [*16j*]	1	O	0.2199(3)	0.3653(3)	0.4430(5)	0.5(1)
Co0.75Ni0.25	O(21) [*16j*]	1	O	0.2178(5)	0.3658(6)	0.4424(5)	0.5(1)
Co0.50Ni0.50	O(21) [*16j*]	1	O	0.2181(5)	0.3654(5)	0.4425(9)	0.5(1)
Co0.25Ni0.75	O(21) [*16j*]	1	O	0.2186(3)	0.3689(5)	0.4411(7)	0.5(1)
Co0Ni1	O(21) [*16j*]	1	O	0.2196(4)	0.3687(4)	0.4419(5)	0.5(1)
Co1Ni0	O(22) [*16j*]	1	O	0.1365(3)	0.5071(5)	0.2136(5)	0.5(1)
Co0.75Ni0.25	O(22) [*16j*]	1	O	0.1368(2)	0.5067(5)	0.2168(4)	0.5(1)
Co0.50Ni0.50	O(22) [*16j*]	1	O	0.1379(7)	0.5059(2)	0.2212(8)	0.5(1)
Co0.25Ni0.75	O(22) [*16j*]	1	O	0.1358(5)	0.5069(3)	0.2129(3)	0.5(1)
Co0Ni1	O(22) [*16j*]	1	O	0.1365(5)	0.5078(4)	0.2134(6)	0.5(1)

Table S3: Main interatomic distances (Å) in the $KCo_{1-x}Ni_xCr_2(PO_4)_3$ compounds.

	Co1Ni0	Co0.75Ni0.25	Co0.50Ni0.50	Co0.25Ni0.75	Co0Ni1
**M(1)−O(11)**	1.988(3) (×4)	2.003(3) (×4)	2.030(2) (×4)	1.979(3) (×4)	1.984(2) (×4)
**M(1)−O(21)**	1.993(3) (×2)	1.979(3) (×2)	1.947(3) (×2)	2.002(2) (×2)	2.000(4) (×2)
**<M(1)−O>**	**1.988(3)**	**1.995(2)**	**2.002(3)**	**1.987(3)**	**1.989(2)**
**BVS**	**2.93**	**2.89**	**2.84**	**2.95**	**2.93**
**M(2)−O(11)**	2.005(3) (×2)	2.013(2) (×2)	2.010(2) (×2)	2.019(2) (×2)	2.011(2) (×2)
**M(2)−O(21)**	2.041(2) (×2)	2.037(2) (×2)	2.040(2) (×2)	2.022(2) (×2)	2.035(2) (×2)
**M(2)−O(22)**	2.058(3) (×2)	2.051(3) (×2)	2.067(3)(×2)	2.046(3)(×2)	2.042(3)(×2)
**<M(2)−O>**	**2.035(4)**	**2.033(2)**	**2.039(2)**	**2.029(3)**	**2.029(4)**
**BVS**	**2.59**	**2.38**	**2.35**	**2.41**	**2.63**
**P(1)−O(11)**	1.541(2) (×2)	1.539 (2) (×2)	1.550(2) (×2)	1.527(2) (×2)	1.547(2) (×2)
**P(1)−O(12)**	1.563(2) (×2)	1.556(2) (×2)	1.562(2) (×2)	1.560(2) (×2)	1.562(2) (×2)
**<P(1)−O>**	**1.552(3)**	**1.548(3)**	**1.556(4)**	**1.544(3)**	**1.555(3)**
**BVS**	**4.77**	**4.82**	**4.72**	**4.88**	**4.73**
**P(2)−O(21)**	1.507(2) (×2)	1.512(2) (×2)	1.513(2) (×2)	1.521(2) (×2)	1.484(2) (×2)
**P(2)−O(22)**	1.562(4) (×2)	1.558(4) (×2)	1.553(4) (×2)	1.560(4) (×2)	1.578(4) (×2)
**<P(2)−O>**	**1.535(3)**	**1.535(3)**	**1.533(4)**	**1.541(3)**	**1.531(3)**
**BVS**	**5.01**	**5.00**	**5.02**	**4.93**	**5.08**
**X(1)−O(11)**	2.642(2) (×2)	2.661(3) (×2)	2.645(4) (×2)	2.631(2) (×2)	2.635(4) (×2)
**X(1)−O(21)**	2.678(3) (×2)	2.670(4) (×2)	2.671(3) (×2)	2.655(3) (×2)	2.638(3) (×2)
**X(1)−O(22)**	2.763(3) (×4)	2.745(2) (×4)	2.742(2) (×4)	2.712(3) (×4)	2.778(2) (×4)
**<X(1)−O>**	**2.712(3)**	**2.705(3)**	**2.700(2)**	**2.678(4)**	**2.707(2)**
**BVS**	**1.68**	**1.70**	**1.73**	**1.83**	**1.72**

Table S4: Crystallite size and microstrain calculation for $KCo_{1-x}Ni_xCr_2(PO_4)_3$ phosphates.

Compounds	D (nm)	Strain G
**Co1Ni0**	177.8	0.050
**Co0.75Ni0.25**	200	0.113
**Co0.50Ni0.50**	189	0.117
**Co0.25Ni0.75**	186	0.115
**Co0Ni1**	202	0.056

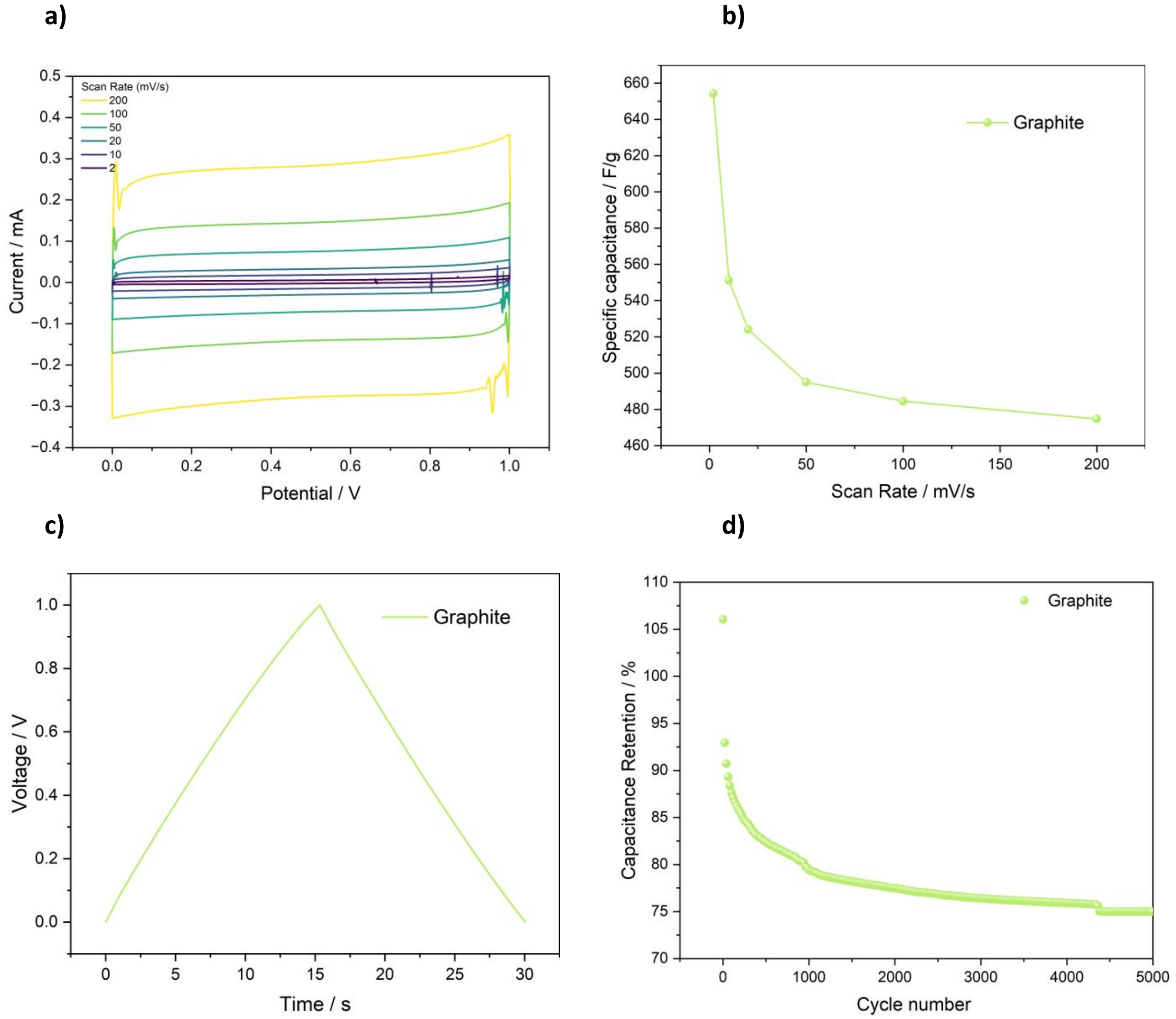

Figure S3: Electrochemical measurements of a pure graphite-based SC device, with (a) CV measurements at different scan rates, (b) specific capacitance values at different scan rates, (c) galvanostatic charge-discharge, and (d) Capacitance retention against cycle number.

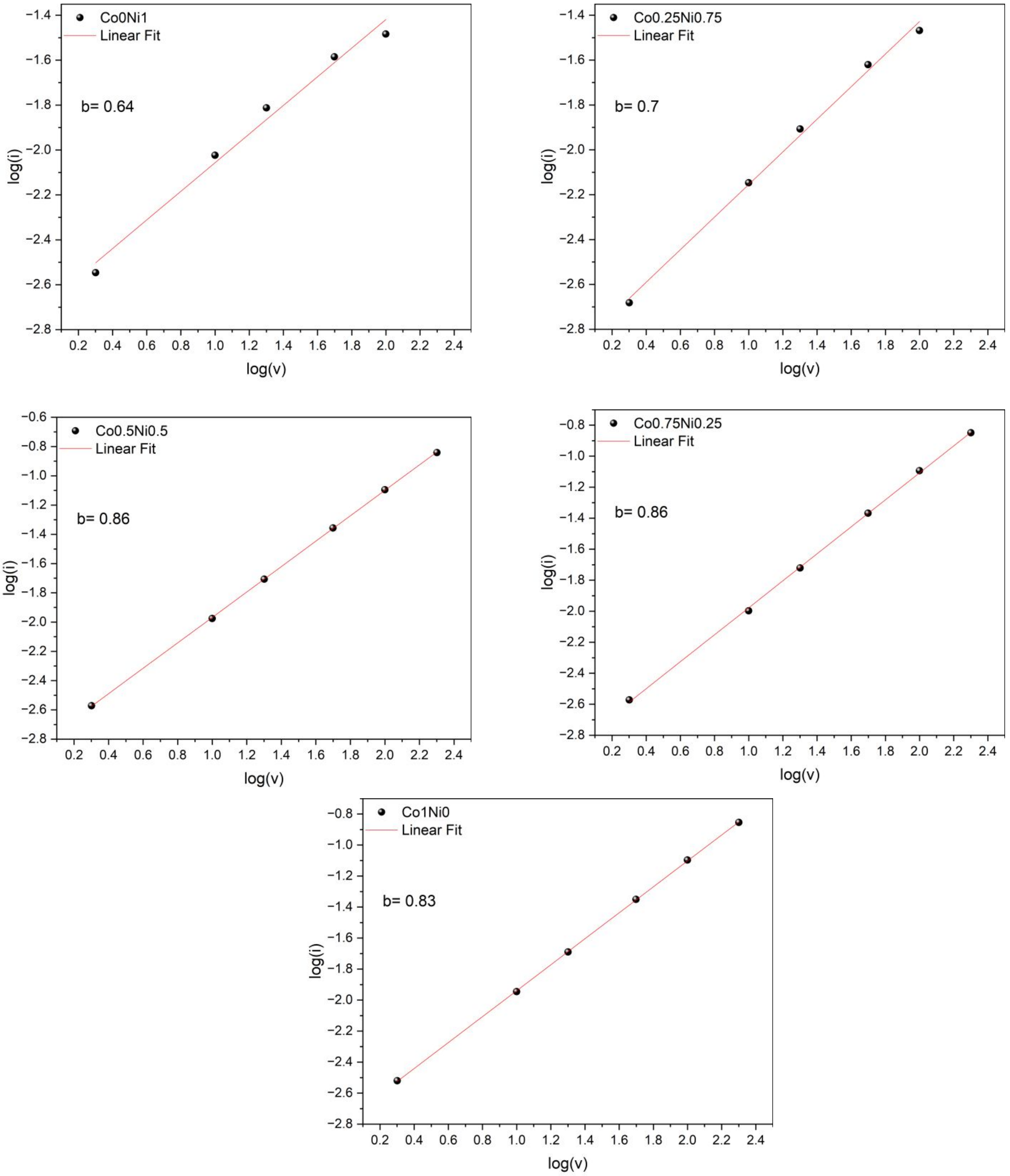

Figure S4: b-value plots of all SC devices.
